# Prediction of metal-free Stoner and Mott-Hubbard magnetism in triangulene-based two-dimensional polymers

**DOI:** 10.1126/sciadv.adq7954

**Published:** 2024-10-02

**Authors:** Hongde Yu, Thomas Heine

**Affiliations:** ^1^ Faculty of Chemistry and Food Chemistry, TU Dresden, Bergstrasse 66c, 01069 Dresden, Germany.; ^2^ Helmholtz-Zentrum Dresden-Rossendorf, Centrum for Advanced Systems Understanding, CASUS, Untermarkt 20, 02826 Görlitz, Germany.; ^3^ Department of Chemistry, Yonsei University and IBS Center for Nanomedicine, Seodaemun-gu, Seoul 120-749, Republic of Korea.

## Abstract

Ferromagnetism and antiferromagnetism require robust long-range magnetic ordering, which typically involves strongly interacting spins localized at transition metal atoms. However, in metal-free systems, the spin orbitals are largely delocalized, and weak coupling between the spins in the lattice hampers long-range ordering. Metal-free magnetism is of fundamental interest to physical sciences, unlocking unprecedented dimensions for strongly correlated materials and biocompatible magnets. Here, we present a strategy to achieve strong coupling between spin centers of planar radical monomers in π-conjugated two-dimensional (2D) polymers and rationally control the orderings. If the π-states in these triangulene-based 2D polymers are half-occupied, then we predict that they are antiferromagnetic Mott-Hubbard insulators. Incorporating a boron or nitrogen heteroatom per monomer results in Stoner ferromagnetism and half-metallicity, with the Fermi level located at spin-polarized Dirac points. An unprecedented antiferromagnetic half-semiconductor is observed in a binary boron-nitrogen–centered 2D polymer. Our findings pioneer Stoner and Mott-Hubbard magnetism emerging in the electronic π-system of crystalline-conjugated 2D polymers.

## Teaser

Ferromagnetic half-metals with spin-polarized Dirac cones and flat bands are predicted in 2D polymers made of radicaloid monomers.

## INTRODUCTION

Long-range magnetic ordering in metal-free materials remains one of the unicorns of physical sciences. While early investigations into magnetic carbon derived from pressurized fullerenes garnered considerable interest, they also raised skepticism; subsequent studies attributed observed phenomena to impurities or defects (*1*). The foundation of metal-free magnetism lies in both stable paramagnetic centers and the ability to control magnetic coupling. Half-filled flat bands in two-dimensional (2D) materials, such as magic-angle twisted bilayer graphene (*2*–*5*), zigzag carbon nanoribbons (*6*, *7*), and 2D polymers with Lieb- or Kagome lattice (*8*–*12*), offer the exploitation of structural topology to generate paramagnetic centers, partially with long-range ordering. These spin-polarized flat bands, with quenched kinetic energy and pronounced electron correlation, harbor a variety of exotic quantum phenomena, ranging from fractional quantum Hall effect (*13*, *14*) and unconventional superconductivity (*5*, *15*, *16*) to high-order topological insulators (*17*), spin frustration (*18*), and Mott insulators (*9*, *19*). However, controlling the magnetic coupling in these systems remains particularly challenging, especially above cryogenic conditions and when aiming for metal-free ferromagnetism (FM).

2D polymers have emerged as a compelling subset of low-dimensional materials, opening avenues for exploration in unconventional electronic and spintronic applications (*12*, *20*, *21*). These polymers, including 2D covalent organic frameworks (COFs) (*22*, *23*), facilitate the precise integration of molecular building blocks into predefined 2D topology. In these architectures, the interplay between chemical composition and lattice topology jointly dominates their electronic properties (*24*–*26*). With the continuous advancement in precision synthesis, 2D polymers of numerous lattices (*8*), such as square (*27*), rectangular (*28*), Lieb (*10*, *11*), honeycomb (*23*), and Kagome (*12*, *24*), have been successfully realized. Despite the variety of structures, attention has predominantly converged on graphene-like 2D polymers with hexagonal symmetry, including honeycomb and Kagome lattices, which arises from their inherent potential to manifest Dirac-like linear band crossings and topological flat bands (*25*, *26*). Although substantial progress has been made (*9*, *17*–*19*, *24*), most of graphene-like 2D polymers are diamagnetic, or those with magnetic centers predominantly exhibit antiferromagnetic (AFM) ordering, which can be primarily attributed to the pronounced electronic coupling within π-orbitals in contrast to the degenerated d-orbitals in inorganic systems (*18*, *29*). Although Lieb’s theorem, also known as the Ovchinnikov rule (*30*), predicts the potential emergence of high-spin polycyclic aromatic hydrocarbons with a nonzero sublattice imbalance, the observation of metal-free FM in extended 2D polymers remains notably rare, especially for systems beyond half-filled π-systems (*11*, *31*).

**Fig. 1.**
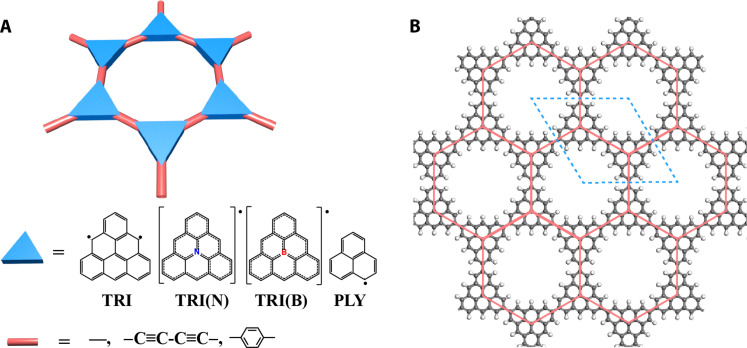
Atomistic structure of triangulenes and its derivatives. Chemical structures of (hetero)TRI monomers and linkages (**A**) and 2D polymer (**B**). The red line in (B) shows the honeycomb lattice, and the blue line illustrates the unit cell.

**Fig. 2.**
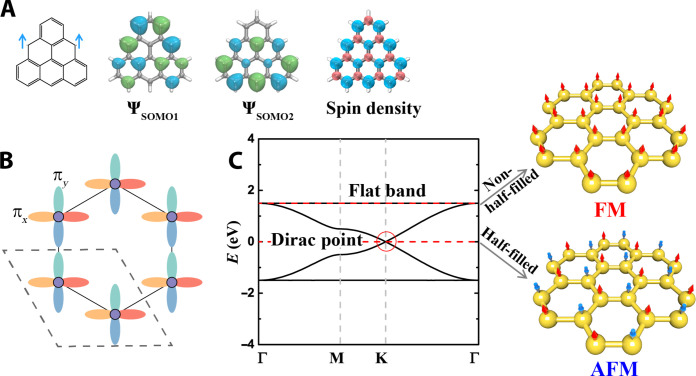
Electronic structure of 2D polymers with dual-orbital honeycomb lattices. (**A**) Chemical structure, the singly occupied molecular orbitals (SOMOs), and the spin density distribution of TRI monomer. Illustrations of honeycomb 2D polymer with degenerate (π*_x_*, π*_y_*) orbitals (**B**) and corresponding band structure (**C**) with Dirac point flanked by two flat bands. The (π*_x_*, π*_y_*) orbitals correspond to the energy-degenerate orthogonal Ψ_SOMO1_ and Ψ_SOMO2_ orbitals of TRI monomer. Upon tuning the electron filling, the systems potentially change from AFM to FM. The red dashed lines indicate the potential Fermi levels upon different filling conditions in various stoichiometric systems.

Triangulene (TRI), characterized by a triplet ground state and non-Kekulé structure, provides a multiradical platform for probing the magnetism in honeycomb 2D polymers (*32*–*34*). In this study, we propose a design concept to control magnetic orderings in 2D polymers with strong magnetic interactions. We investigate the metal-free magnetism in 15 TRI-based 2D polymers (Fig. 1). Using both the tight-binding (TB) method and first-principles calculations, we find the distinctive band structure of these 2D polymers for the hypothetical spin-unpolarized states, which is characterized by a Dirac point enveloped by two flat bands (Fig. 2). This characteristic of the dual-orbital honeycomb lattice, in contrast to graphene and many other monoradical-based 2D polymers, stems from the doubly degenerate frontier orbitals of TRI monomer. For half-filled systems, such as pristine carbon–centered and boron-nitrogen (BN)–centered binary TRI 2D polymers, the electron correlation opens bandgaps at the Dirac points, resulting in Mott-Hubbard insulators with AFM ground state.

Contrarily, for non–half-filled systems, the electron correlation and large density of states near the Fermi level lead to metal-free Stoner FM, where opposite spin channels shift to different energies. We observe strong magnetic couplings of 59 and 56 meV in nitrogen- and boron-centered TRI [TRI(N) and TRI(B), respectively] 2D polymers (i.e., aza- and bora-TRI), corresponding to Curie temperatures of 260 and 250 K, respectively. This FM ground state is beyond the predictive power of Lieb’s theorem. We further demonstrate that these FM systems are half-metals, where one spin channel shows a linear band crossing with an associated “massless” spin character, whereas the opposing channel behaves as a single-band semiconductor. This unique feature offers potential application as spin filters to accommodate massless spin currents in organic spintronics. In contrast to AFM Mott insulators without spin splitting, AFM-coupled BN-centered binary 2D polymers are half-semiconductors, where spin channels have different bandgaps, arising from the variation of chemical potential. This system, simultaneously having both spin splitting and fully compensated spins, offers new opportunities for manipulating spin conduction through an external electric field. Our work elucidates innovative avenues for realizing FM in organic magnets, specifically via spin-polarized flat bands, thus paving the way for groundbreaking device applications in forthcoming research.

## RESULTS

### TRI-based 2D polymers: Dual-orbital honeycomb lattices

TRI, first proposed by Clar and Stewart in 1953 (*35*), is recognized as the smallest nanographene featuring a triplet ground state. This biradical with a unique non-Kekulé structure has been synthesized both in solution and on the surface (Fig. 1A) (*33*, *34*, *36*). In addition to pristine carbon–centered TRI, hetero-TRIs, such as TRI(N) (i.e., aza-TRI) (*37*–*40*) and TRI(B) (i.e., bora-TRI) (*41*), and smaller variant phenalenyl (PLY) (i.e., [2] TRI) (*42*–*44*) have also been realized because of rapid advancements in precision synthesis of planar organic radicals (Fig. 1). Notably, the TRI monomer has two energy-degenerate orthogonal radical orbitals [i.e., singly occupied molecular orbitals (SOMOs)] (Fig. 2A) (*34*), differing from many monoradicals with a single SOMO, such as PLY, perchlorotriphenylmethyl (PTM), triarylmethyl (TAM), and trioxotriangulene (TOT) (*9*, *19*). In all these systems, the SOMOs expand over the molecular plane. These triangular-shaped monomers can be integrated into 2D polymers via well-established Yamamoto or Ullmann coupling reactions (*32*, *33*, *45*). Here, we create models of 15 2D polymers, using these radicals as building blocks, linked directly or bridged by common linear linkages, including ─C≡C─C≡C─ (CCCC) and phenyl (Ph) (Fig. 1 and figs. S1 to S5).

As shown in Figs. 1B and 2D, polymers made of TRI and its aforementioned derivatives form a honeycomb lattice similar to graphene (Fig. 1B and fig. S6) (*46*). However, unlike graphene and many monoradical-based 2D polymers with only one half-filled orbital per lattice site, such as PLY, TOT, TAM, and PTM (*9*, *47*), the TRI-based 2D polymers host two orthogonal orbitals at each site (denoted as π*_x_* and π*_y_*), originating from the degenerate SOMO orbitals of its triplet monomer (Fig. 2A). We first explore the electronic structure of this four-orbital–based 2D lattice using a TB model with an effective (π*_x_*, π*_y_*) basis and the Slater-Koster scheme (*48*). The corresponding effective Hamiltonian representing single-electron properties is expressed as

$$H_{\text{eff}}=\left(\begin{array}{cccc} \varepsilon_{0} & 0 & V_{\mathrm{xx}} & V_{\mathrm{xy}}\\ 0 & \varepsilon_{0} & V_{\mathrm{xy}} & V_{\mathrm{yy}}\\ V_{\mathrm{xx}}^{*} & V_{\mathrm{xy}}^{*} & \varepsilon_{0} & 0\\ V_{\mathrm{xy}}^{*} & V_{\mathrm{yy}}^{*} & 0 & \varepsilon_{0} \end{array}\right)$$

where $V_{\mathrm{xx}}=\left(\frac{3}{4}\mathrm{pp}\sigma +\frac{1}{4}\mathrm{pp}\pi \right)\left(e^{ika_{1}}+e^{ika_{2}}\right)+\mathrm{pp\pi}\;e^{ik\left(a_{1}+a_{2}\right)}$, $V_{\mathrm{yy}}=\left(\frac{1}{4}\mathrm{pp}\sigma +\frac{3}{4}\mathrm{pp}\pi \right)\left(e^{ika_{1}}+e^{ika_{2}}\right)+\mathrm{pp}\sigma \;e^{ik\left(a_{1}+a_{2}\right)}$, $V_{\mathrm{xy}}=\frac{\sqrt{3}}{4}\left(\mathrm{pp}\sigma -\mathrm{pp}\pi \right)\left(e^{ika_{1}}-e^{ika_{2}}\right)$, *pp*σ, and *pp*π are the Slater-Koster parameters (*25*, *26*).

As illustrated by Fig. 2 (B and C), the (π*_x_*, π*_y_*)-TB model, representing the 2D polymers of TRIs with doubly degenerate frontier orbitals, yields a distinctive four-band structure: a Dirac point flanked by two flat bands. In contrast, graphene only shows a Dirac point near the Fermi level (*47*). This four-orbital honeycomb lattice is different from many typical hexagonal lattices, such as one-orbital honeycomb, Kagome lattice, and honeycomb-Kagome lattice (figs. S7 to S9) (*8*, *49*). Such an unconventional electronic structure stems from the number of on-site orbitals and has seldom been observed in monoradical-based 2D polymers, such as those made of TOT, TAM, and PTM (figs. S10 to S12) (*9*). Beyond TRI-based 2D polymers, this (π*_x_*, π*_y_*)-TB model can also represent many 2D polymers structured with a honeycomb lattice and composed of monomers with doubly degenerate orbitals. For instance, it can delineate the band structure of diamagnetic 2D COF with benzene nodes that also has energy-degenerate highest occupied molecular orbitals (fig. S13) (*26*).

**Fig. 3.**
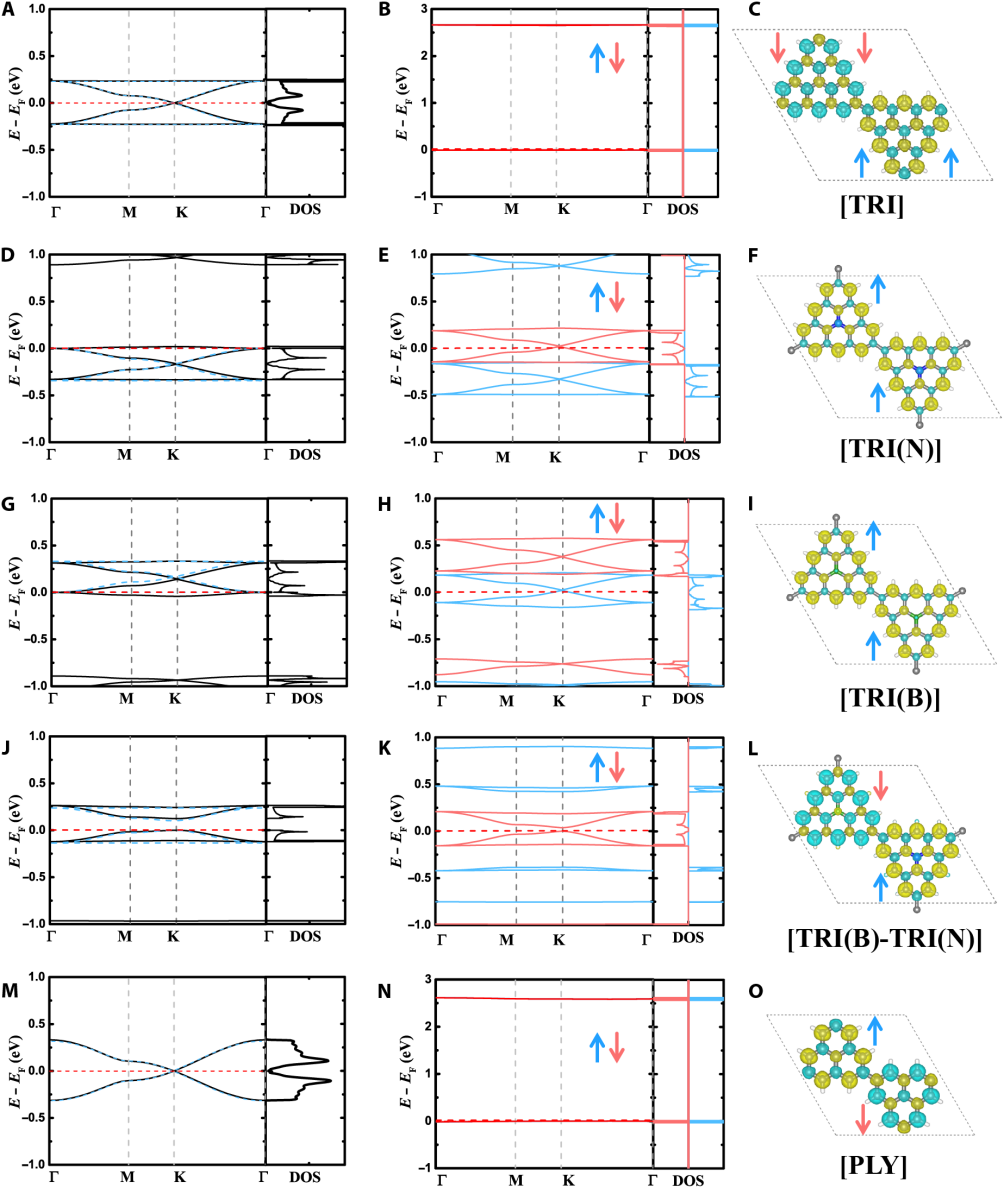
Electronic structures of TRI-based 2D polymers. Band structure and density of states (DOS) of diamagnetic (**A**, **D**, **G**, **J**, and **M**) and spin-polarized (**B**, **E**, **H**, **K**, and **N**) ground states and spin density distribution (**C**, **F**, **I**, **L**, and **O**) of TRI-based 2D polymers, including [TRI] [(A) to (C)], [TRI(N)] [(D) to (F)], [TRI(B)] [(G) to (I)], [TRI(B)-TRI(N)] [(J) to (L)], and [PLY] [(M) to (O)]. Blue dashed lines in diamagnetic band structures are calculated by the TB model. For [TRI], [PLY], and [TRI(B)-TRI(N)], the ground states are AFM states, while they are FM states for [TRI(N)] and [TRI(B)]. [TRI(N)], [TRI(B)], and [TRI(B)-TRI(N)] are calculated with Perdew-Burke-Ernzerhof (PBE) functional, while [TRI] and [PLY] with PBE0 functional (see Materials and Methods, figs. S29 to S31, and table S2 for more details).

Under half-filling conditions, the Fermi level of carbon-centered TRI 2D polymer, [TRI], intersects the Dirac point resembling graphene (Figs. 2C and 3A). Conversely, by adjusting the electron number of the system, it is feasible to shift the Fermi level to align with the flat band, for example, via introducing nitrogen or boron in [TRI(N)] and [TRI(B)]. Within the flat bands, the many-body effects typically dominate over the kinetic energy, resulting in high density of states for strongly correlated electrons due to the interference effect (*50*), despite the presence of substantial orbital overlap (Figs. 2C and 3D). This differs from the atomic flat bands observed at the core level or in the presence of dangling bonds, which originate from the lack of overlap between adjacent atomic orbitals (*50*). These flat bands with extended electronic states and pronounced electron correlation can evoke many intriguing physical phenomena, such as the fractional quantum Hall effect and unconventional superconductivity, as observed in twisted bilayer graphene and 2D Kagome lattices (*5*, *51*). In addition, the high density of states near the Fermi level corresponding to the flat bands could induce spontaneous spin polarization according to the Stoner model (*52*), where *U*/*t* > 1 is a critical value for phase transition. However, in most metal-free systems, introducing spin polarization is of fundamental challenge due to the absence of degenerate orbitals coupled with the presence of strong electronic coupling (*t*) in the π-orbitals. This contrasts sharply with inorganic compounds featuring transition metals and energy-degenerate d-orbitals. (Multi)radicaloids, such as TRIs, provide degenerate π-orbitals as spin centers, analogous to the role of atoms with degenerate d-orbitals in inorganic magnets. Larger TRIs, also known as graphene nanoflakes, have more parallel spins within their finite structures, in accordance with Hund’s rule as applied to incompletely filled d- and f-orbitals (*53*, *54*). However, to create solids with long-range magnetic ordering, it is vital to connect these “superatoms” (i.e., TRI monomers) via chemical bonds into extended periodic structures as in 2D polymers and to control the magnetic interaction between them.

### Spin polarization and magnetic interaction in TRI-based 2D polymers

As carbon-centered TRI 2D polymers, including [TRI] and [PLY], have balanced sublattices, Ovchinnikov’s rule is applicable and predicts that they have a singlet ground state, i.e., *S* = 0 (figs. S14 to S17). From a classical physical chemistry viewpoint, the binary 2D polymer [TRI(B)-TRI(N)] is isoelectronic to [TRI]. However, the different chemical potential of the monomers in [TRI(B)-TRI(N)] will result in a unique electronic structure. However, it is unclear whether it is a closed-shell (diamagnetic) or open-shell (AFM) singlet state. To understand the ground state and the spin polarization in these polymers, we used first-principles calculations as implemented in Vienna Ab initio Simulation Package (VASP 5.4.4) (*55*) and CRYSTAL17 (*56*) (see Materials and Methods for computational details). As illustrated in Fig. 3A, for the diamagnetic state, [TRI] shows a band structure featuring a Dirac point and twin flat bands, which can be well represented by the four-orbital TB model with a fitted *t* = 0.16 eV. As in graphene, its Fermi level intersects the Dirac point (Fig. 3A). However, unlike graphene’s diamagnetic and semimetallic ground state, the spin-polarized singlet state in [TRI] is by 2.17 eV more stable than the diamagnetic one. This is in accord with the non-Kekulé structure and the corresponding unpaired electrons featured by its radical building blocks. This is further manifested by a substantial *U*/*t* ratio of 16.6 as per the Stoner model (Table 1). Hereafter, to evaluate the stability of spin-polarized states, the spin polarization energy (Δ*E*_spin_) is defined by the energy difference between spin-polarized ground state (*E*_GS_) and diamagnetic state (*E*_CS_), Δ*E*_spin_ = *E*_GS_ − *E*_CS_.

**Table 1.** Magnetic properties of the TRI-based 2D polymers. It includes magnetic coupling (*J*), the magnetic moment per unit cell (*M*), electronic coupling (|*t*|), on-site Coulomb repulsion (*U*), *U*/*t*, and spin polarization energy (Δ*E*_spin_). [TRI(N)], [TRI(B)], and [TRI(B)-TRI(N)] are calculated with PBE functional, while [TRI] and [PLY] are with PBE0 functional (see Materials and Methods and table S2 for more details).

	*J* (meV)	*M* (μB)	*U* (eV)	|*t*| (eV)	*U*/*t*	Δ*E*_spin_ (eV)
[TRI]	−31.77	0.00	2.66	0.16	16.6	−2.17
[TRI(N)]	59.20	2.00	0.35	0.01	35.00	−0.09
[TRI(B)]	55.90	2.00	0.38	0.01	38.00	−0.09
[TRI(B)-TRI(N)]	−124.80	0.00	0.27	0.12	2.25	−0.07
[PLY]	−97.56	0.00	2.61	0.11	23.7	−1.04

**Fig. 4.**
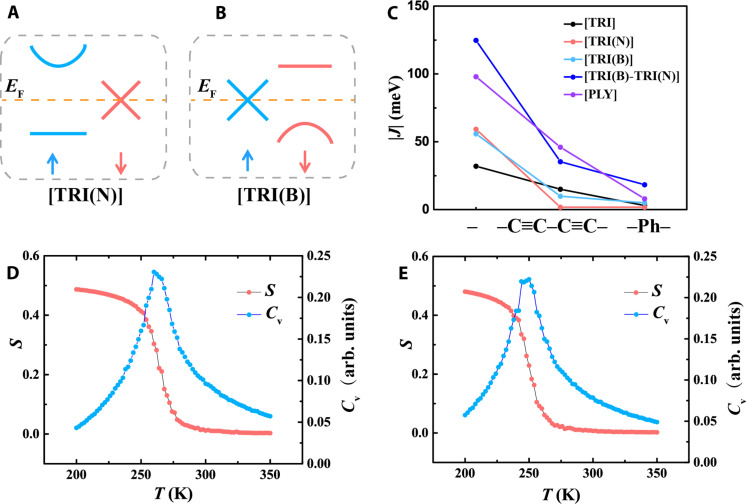
FM half-metallic frontier bands and corresponding Curie temperature. Illustration of half-metallic band structures of [TRI(N)] (**A**) and [TRI(B)] (**B**). (**C**) Correlations between linkages and strength of magnetic coupling (|*J*|). Normalized spin (*S*) per site and electronic heat capacity (*C*_v_) with the temperature from the Monte Carlo (MC) simulations of [TRI(N)] (**D**) and [TRI(B)] (**E**). arb. units, arbitrary units.

We further find for [TRI] that the AFM state is more stable than the FM state, with a magnetic coupling of −32 meV between adjacent sites, according to the Heisenberg–Dirac–van Vleck Hamiltonian, $\hat{H}=-\sum_{<i,j>}J{\hat{S}}_{i}{\hat{S}}_{j}$ (Fig. 3, B and C, and Table 1). From a spin exchange perspective, the strong electronic coupling of 0.16 eV between neighboring sites results in a predominant kinetic exchange of −38 meV, favoring AFM interaction and antiparallel spin alignment (Table 1). As a consequence of the pronounced on-site Coulomb repulsion, [TRI] becomes a Mott insulator with a bandgap of 2.66 eV, thus diverging from the “gapless” semimetallic nature of graphene (Fig. 3B). Indicated by the degenerate AFM bands of [TRI], the spin-up and spin-down electrons are energetically equivalent and delocalized in momentum space, whereas, in real space, they are highly localized within adjacent monomers owing to the on-site Coulomb repulsion (Fig. 3, B and C). Similar phenomena are also observed in metal-oxide complexes and 2D polymers made of monoradicals, such as monolayer PTM-COF, TOT-COF, and TAM-COF (*9*, *19*, *52*). Analogously, other half-filled 2D polymers, including [PLY] and [TRI(B)-TRI(N)], obey Ovchinnikov’s rule and exhibit AFM ground states with *J* values of −98 and −125 meV. These *J* values substantially exceed the Landauer limit for minimum energy dissipation (18 meV), indicating their potential as promising candidates for spin logic operations at room temperature (*57*). In addition, the magnetic coupling *J* decreases as the spacer lengthens from direct linkage to ─CCCC─ and ─Ph─, arising from the reduction in the overlap between localized spin orbitals (Fig. 4C and figs. S18 to S27).

Besides Ovchinnikov’s rule, for a spin-polarized system, the Goodenough-Kanamori rule can be used to predict and understand the magnetic interactions by analyzing the angle and type of adjacent spin-polarized orbitals. In general, within the flat bands, the limited dispersion (*t* ≈ 0) will neutralize the superexchange interaction (−4*t*^2^/*U*), resulting in “orthogonal” extended wave functions (*58*). Therefore, the potential exchange, favoring parallel spin alignments, prevails, leading to flat-band FM. Despite substantial observations of flat bands in diamagnetic Kagome 2D polymers, reports on metal-free FM are still very rare (*31*, *59*).

Different from half-filled TRI 2D polymers, such as [TRI] and [PLY], using electron-sufficient building units as in [TRI(N)] will introduce additional π-electrons to the systems and elevate the Fermi level to the flat bands, while maintaining the stoichiometric and charge neutrality of the system (*24*). The corresponding high density of states near the Fermi level stimulates spontaneous spin polarization, as demonstrated by the large *U*/*t* ratio of 35, according to the Stoner model (Fig. 3, D and E, and Table 1). Furthermore, the FM state is energetically more stable than the AFM state with a *J* value of 59 meV, resulting in parallel spin alignment (Table 1 and Fig. 3F). This metal-free FM and corresponding high-spin ground state appears to be beyond the scope of Ovchinnikov’s rule. However, this behavior can be understood from the Goodenough-Kanamori rule. Since the kinetic exchange −4*t*^2^/*U*, stemming from the virtual hopping of antiparallel spins, is suppressed by the limited band dispersion of the flat band (*t* = 0.01 eV), the direct exchange (i.e., potential exchange) between neighboring parallel spins governs the magnetic interaction, leading to the overall FM coupling (Table 1). Similarly, 2D polymers made of the electron-deficient TRI(B) monomer, i.e., [TRI(B)], shift the Fermi level down to the flat band as a π-electron per monomer is diminished compared to [TRI] (Fig. 3G). Consequently, [TRI(B)] also emerges as a 2D FM polymer with a magnetic coupling of 56 meV, arising from the reduced electronic coupling of 0.01 eV and the kinetic exchange of −1 meV (Table 1). For both [TRI(N)] and [TRI(B)], the FM ground states are lower than the AFM states, which, in turn, are more stable compared to the diamagnetic configurations (see table S1).

The Curie temperatures *T*_c_ for [TRI(N)] and [TRI(B)] are predicted using Monte Carlo (MC) simulations, demonstrating spontaneous FM phase transitions occurring at about 260 and 250 K, respectively. This indicates the possibility of forming long-range parallel spin ordering near room temperature (Figs. 3C and 4, D and E). These *T*_c_ surpass many inorganic 2D van der Waals FM systems, such as Cr_2_Ge_2_Te_6_ (*J* ≈ 4 meV, *T*_c_ = 66 K) and CrI_3_ (*J* ≈ 4 meV, *T*_c_ = 45 K), which can be ascribed to the stronger magnetic coupling in these 2D polymers with spin-polarized flat bands (*60*, *61*). We notice the long-standing controversy about the existence of magnetism in low-dimensional systems (*62*), especially for the metal-free systems featuring small spin-orbit coupling and magnetic anisotropic energy, as the Mermin-Wagner theorem prevents the long-range magnetic order at any finite temperature as consequences of thermal fluctuations (*63*). However, long-range spin order has been observed even in 1D systems, such as monatomic metal chains of cobalt and zigzag edges of narrow graphene nanoribbon (*64*, *65*), in contrast to the Ising model. Recent research shows that, at finite system size that is over several millimeters and exceeds the scale of typical nanotechnology devices, magnetic ordering can be stabilized by short-ranged interactions without any magnetic anisotropy, although for infinite-size systems, it will be disturbed at nonzero temperature (*66*). Our findings demonstrate that the topological flat band at the Fermi level and metal-free FM can be achieved by manipulation of topology and chemical components in the non-Kekulé 2D polymers without the need for external doping or high magnetic fields. Note that metal surfaces, often serving as the support for these 2D polymers, can affect the direct measurement of intrinsic properties. Their strong coupling with the 2D polymers can result in charge transfer and trigger the Kondo effect. For example, the charge transfer between the metal surface and 2D polymers may quench or enhance the magnetic moments due to the difference in the Fermi level of metal and polymer (*37*). However, several approaches have been proposed to decouple the metal surface from the 2D polymers, including the use of a semiconducting substrate or an intercalating layer (*67*, *68*).

### Spin conduction in TRI-based 2D polymers

In contrast to the AFM Mott insulators of carbon-centered TRI 2D polymers and the semimetal behavior of graphene, both [TRI(N)] and [TRI(B)] distinctly exhibit FM half-metallic properties, where one spin channel is (semi)metallic and the other is single-band semiconductor (Figs. 3, E and H, and 4, A and B). For [TRI(N)], the Fermi level intersects the Dirac point of the spin-down channel, indicating massless spin-down electrons, where the wave function is fully extended (Figs. 3E and 4A and fig. S28). As the FM breaks the time-reversal symmetry and two spin-polarized bands have a linear crossing, these 2D polymers should host Weyl fermions in comparison to Dirac fermions that typically have four-component spinors and time-reversal symmetry (*69*). Further validation of the emergence of Weyl fermions will be investigated in future work. Conversely, the spin-up channel shows unusual spin-polarized single-band semiconducting characteristics with a bandgap of 0.96 eV (Figs. 3E and 4A). This single-band feature provides an opportunity to disentangle the motions of charge carriers, due to the mobile electrons at the parabolic conduction band maximum and the heavy holes at the flat valence band. [TRI(B)] also demonstrates a semimetallic feature for the spin-up channel, while the spin-down channel is semiconducting with a bandgap of 0.94 eV (Figs. 3H and 4B). These characteristics imply that [TRI(N)] and [TRI(B)] can potentially serve as spin filters in organic spintronics for spin-down and spin-up electrons, respectively (*70*, *71*). Moreover, because of the linear dispersion at the Dirac point, spin-polarized currents can be transported with remarkably high mobility. Unlike inorganic systems containing heavy elements, organic materials composed of B, C, and N atoms generally have smaller spin-orbit coupling, which impedes spin relaxation, thereby maintaining spin polarization over a larger diffusion length and longer relaxation time (*18*, *72*, *73*).

The binary 2D polymer, [TRI(B)-TRI(N)], composed of alternating TRI(N) and TRI(B) monomers, is also at half-filling, similar to its isoelectronic counterpart [TRI] (Fig. 3J). However, distinct from [TRI], the different chemical potential in TRI(B) and TRI(N) induces a bandgap opening of 0.12 eV at the Dirac point for the diamagnetic state (Fig. 3J). This behavior parallels that observed in hexagonal boron nitride relative to graphene and can be recapitulated by the TB model with an on-site energy difference of 0.1 eV (Fig. 3J). [TRI(B)-TRI(N)] also exhibits an AFM ground state as per Ovchinnikov’s rule analogous to [TRI] (Table 1 and Fig. 3L). Different from most AFM 2D polymers with degenerate spin-up and spin-down bands, for example, [TRI], [PLY], PTM-COF, TOT-COF, and TAM-COF (*9*), [TRI(B)-TRI(N)] is a spin-polarized half-semiconductor. In particular, [TRI(B)-TRI(N)] shows a narrow bandgap of 0.037 eV for spin-down electrons and a substantially larger one of 0.81 eV for spin-up electrons (Fig. 3K). This unprecedented half-semiconductor property in 2D polymers offers immense possibilities for controlling spin conductance through external fields. For instance, the application of laser light of a specific wavelength or an electric field could lead to the generation of a spin-polarized current via excitation or injection.

## DISCUSSION

In summary, we proposed a spin-polarized flat band approach to realizing metal-free FM in 2D polymers. Using first-principles calculations, we examined 15 TRI-based 2D polymers, where a distinct sandwiched electronic structure with a Dirac point and twin flat bands was unveiled. We demonstrated that half-filled systems, such as [TRI], [PLY], and [TRI(B)-TRI(N)], had AFM magnetic couplings of −32, −98, and −125 meV, respectively. Beyond half-filling, [TRI(N)] and [TRI(B)] showed metal-free FM with *J* values of 59 and 56 meV, respectively. These FM couplings were due to the intersection of the Fermi level and the flat band, suppressing the electronic coupling and the AFM kinetic exchange, as per the Goodenough-Kanamori rule. MC simulations predicted Curie temperatures of 260 and 250 K for these systems, implying the stability of long-range spin ordering at near room temperatures. Compared to the AFM Mott insulators of [TRI] and [PLY], [TRI(N)] and [TRI(B)] were half-metals. In these 2D polymers, one spin channel was semimetallic owing to the linear band crossing at the Fermi level, while the other spin channel was a single-band semiconductor. In addition, we presented unprecedented AFM half-semiconductor properties in [TRI(B)-TRI(N)], where spin-polarized bands are energetically nondegenerate. These spin-dependent bandgaps furnish considerable opportunities for the fine-tuning and control of spin-conducting behavior via external fields. Our findings not only enrich the fundamental understanding of the electronic and magnetic properties of 2D polymers but also provide promising candidates for the realization of metal-free FM and advancement of organic spintronics, particularly as spin filters or generators. Note that after submission of our manuscript, similar results on [TRI(N)] have been reported on the basis of the mean-field Hubbard method by Fernández-Rossier and colleagues (*74*).

## MATERIALS AND METHODS

### Density functional theory calculations

The lattice parameters and atomic coordinates of all the 2D polymers were optimized using the projector augmented wave (*75*) method and the Perdew-Burke-Ernzerhof (PBE) (*76*) exchange-correlation functional as implemented in the Vienna Ab initio Simulation Package (VASP 5.4.4) (*55*). We applied Grimme’s D3 approach for London dispersion correction (*77*). Local density approximation (LDA) or generalized gradient approximation (GGA) functionals, such as PBE, are suitable to predict the magnetic properties of metals and small gap semiconductors, where itinerant character is prominent. In contrast, hybrid functionals, such as PBE0 and Heyd–Scuseria–Ernzerhof (HSE06), can be used to study Mott insulators, where the electrons are highly localized (*78*–*80*). Therefore, we used PBE functional to investigate [TRI(N)], [TRI(B)], and [TRI(B)-TRI(N)] and PBE0 functional for [TRI] and [PLY]. However, note that the band structures and magnetic properties calculated by PBE, PBE0, and HSE06 functionals are very similar in these 2D polymers, as demonstrated in figs. S29 to S31 and table S2. Predicting the magnetic coupling with quantitative accuracy for a periodic system is a fundamental challenge, as it requires the correct integration of the system’s multireference character in the calculation. The restricted open-shell Kohn-Sham (ROKS) approach is an alternative to describe the open-shell singlet excited state (*81*–*83*) and to predict charge transfer and core excitations (*84*, *85*). It performs the self-consistent field (SCF) procedure simultaneously on both the “mixed” singlet and triplet states and removes the spin contamination of the open-shell singlet (OSS) state, which is impossible in broken-symmetry (BS)–density functional theory (DFT), marking its principal deficiency. For [TRI(N)] and [TRI(B)], we calculated the OSS state with the ROKS method as implemented in the CP2K package (*86*) and derived the magnetic coupling (table S3). For these calculations, we used the PBE functional with the double-zeta basis set (DZVP-MOLOPT) basis set and the Goedecker-Teter-Hutter pseudo-potential. The orbital transformation method was used with a supercell of 3 × 3 × 1, which maps the *K* point to Γ. The *J* values of 66 and 69 meV produced by the ROKS method are slightly larger than those of BS-DFT (59 and 56 meV, respectively). In our previous work, we demonstrated a high-spin ground state for the TRI(N) dimer at a coplanar configuration (*87*). We used a cutoff energy of 400 eV for the plane-wave basis set for structure optimizations and 600 eV for static calculations. We set the convergence criterion for forces on atoms during optimizations to 0.005 eV/Å, while the energy convergence criterion in the SCF iteration was set to 10^−5^ eV for optimizations and 10^−6^ eV for static calculations to obtain the converge spin and charge density. The *k*-mesh of 8 × 8 × 1 was used for both optimization and static calculations, while 15 × 15 × 1 was used to obtain the density of states. For PBE0 and HSE06 calculations, we used POB-TZVP basis set as implemented in the CRYSTAL17 software (*56*). The *k*-mesh of 16 × 16 × 1 was used in the SCF processes. As only C, N, and B elements are in these systems, spin-orbit coupling was not considered in the calculations. For [TRI(N)], our calculations show that spin-orbit coupling can only open a gap at 10^−4^ to 10^−5^ eV, which is beyond the numerical accuracy of DFT calculations. The magnetic coupling *J* was calculated by the normalized energy difference between AFM and FM states, as defined by *J* = (*E*_AFM_ − *E*_FM_)/2*zS*^2^, where *z* is the number of nearest neighbors, i.e., 3 for honeycomb lattices, and *S* is the spin on each site, i.e., 1 for TRI and 1/2 for TRI(N), TRI(B), and PLY. To evaluate the stability of spin-polarized states, the spin polarization energy was calculated by the energy difference between the spin-polarized ground state (*E*_GS_) and closed-shell state (*E*_CS_), as defined by Δ*E*_spin_ = *E*_GS_ − *E*_CS_, where GS is FM state for [TRI(N)] and [TRI(B)], while it is AFM state for [TRI], [TRI(B)-TRI(N)], and [PLY]. The electronic coupling (intersite hopping integral), *t*, was calculated by fitting the band structure calculated by DFT with TB model. The on-site Coulomb repulsion *U* was calculated by the energy difference between spin-up and spin-down bands. Multiwfn was used for the visualization of wave functions (*88*). The optimized structures and other data necessary to reproduce our results were provided in Zenodo repository (*89*).

### TB calculations

TB model within a (π*_x_*, π*_y_*) basis was used to describe the electronic structures of TRI-based 2D polymers, where the *pp*σ = 1 eV and *pp*π = 0 are used in Fig. 2C for illustration and *pp*σ = |*t*| and *pp*π = 0 are used in fig. S14 for fitting. Arising from the variation of chemical potential of TRI(N) and TRI(B), the difference of on-site energy was set to 0.1 eV in [TRI(B)-TRI(N)], while, for other 2D polymer, the site energy is 0 eV. For [PLY], TB model with single orbital on each site was applied. We used Pybinding package to solve the TB model.

### MC simulations

We used MC simulations with metropolis algorithm to determine the Curie temperature of [TRI(N)] and [TRI(B)]. The specific electronic heat capacity (*C*_v_) was calculated by $C_{v}=\frac{<E^{2}>-<E{>}^{2}}{k_{B}T^{2}}$. We used 40 × 40 × 1 supercell in the simulation and calculated 50 trajectories at every temperature. For each trajectory, we calculated 50,000,000 steps, while the last 5,000,000 steps are used to average and generate the data.
