# Supplementary material for: Prediction of metal-free Stoner and Mott-Hubbard magnetism in triangulene-based two-dimensional polymers
Source: Sci Adv. 2024 Oct 2;10(40):eadq7954. doi: 10.1126/sciadv.adq7954 (PMC12697527; doi:10.1126/sciadv.adq7954)
Supplement: Supplementary file 1 — Figs. S1 to S31 Tables S1 to S3 [file sciadv.adq7954_sm.pdf]

Science Advances
AAAS

Supplementary Materials for

Prediction of metal-free Stoner and Mott-Hubbard magnetism in triangulene-based two-dimensional polymers

Hongde Yu and Thomas Heine

Corresponding author: Thomas Heine, thomas.heine@tu-dresden.de

Sci. Adv. **10**, eadq7954 (2024)
DOI: 10.1126/sciadv.adq7954

This PDF file includes:

Figs. S1 to S31
Tables S1 to S3

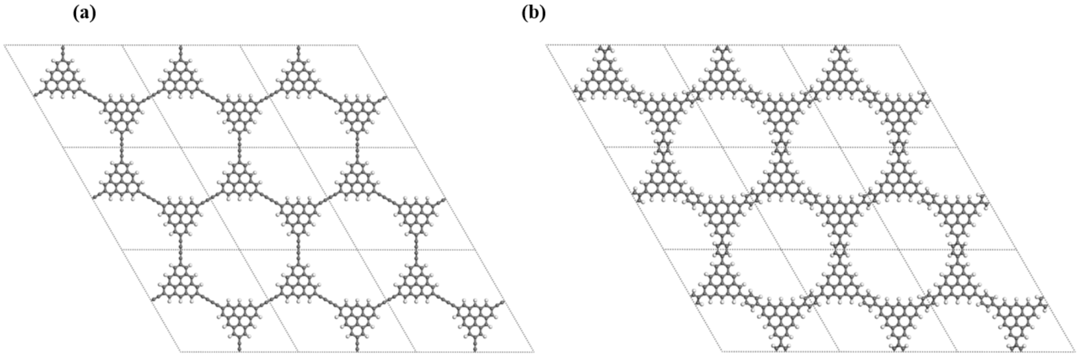

Fig. S1. Chemical structures of [TRI-CCCC] and [TRI-Ph]. (A) and (B) correspond to [TRI-CCCC] and [TRI-Ph], respectively.

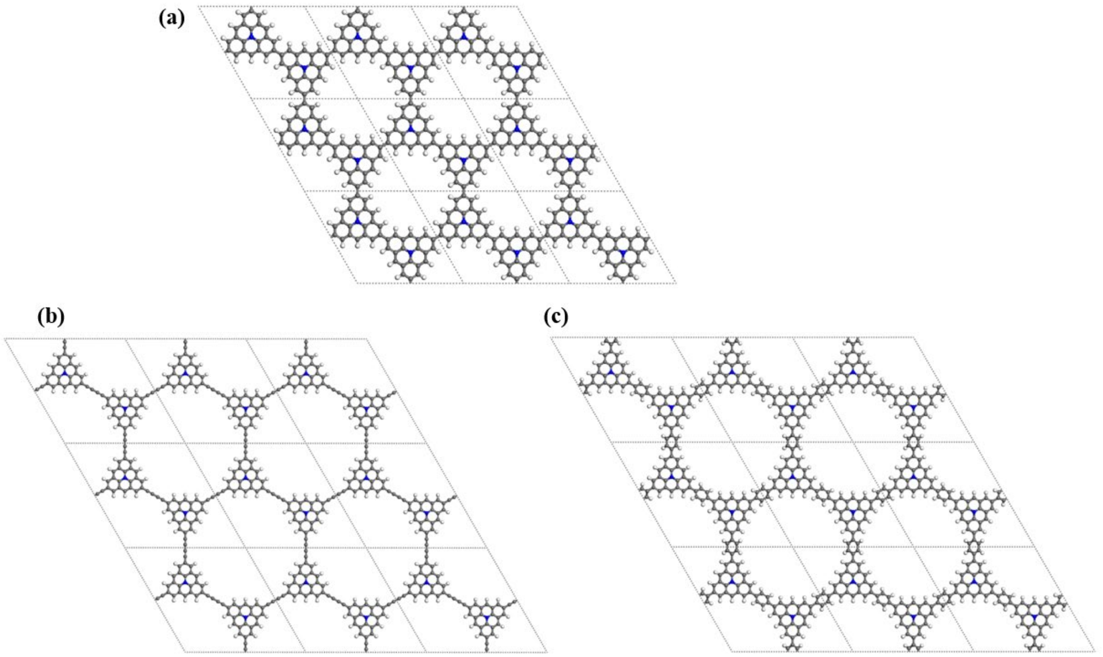

Fig. S2. Chemical structures of [TRI(N)], [TRI(N)-CCCC] and [TRI(N)-Ph]. (A)-(C) correspond to [TRI(N)], [TRI(N)-CCCC] and [TRI(N)-Ph], respectively.

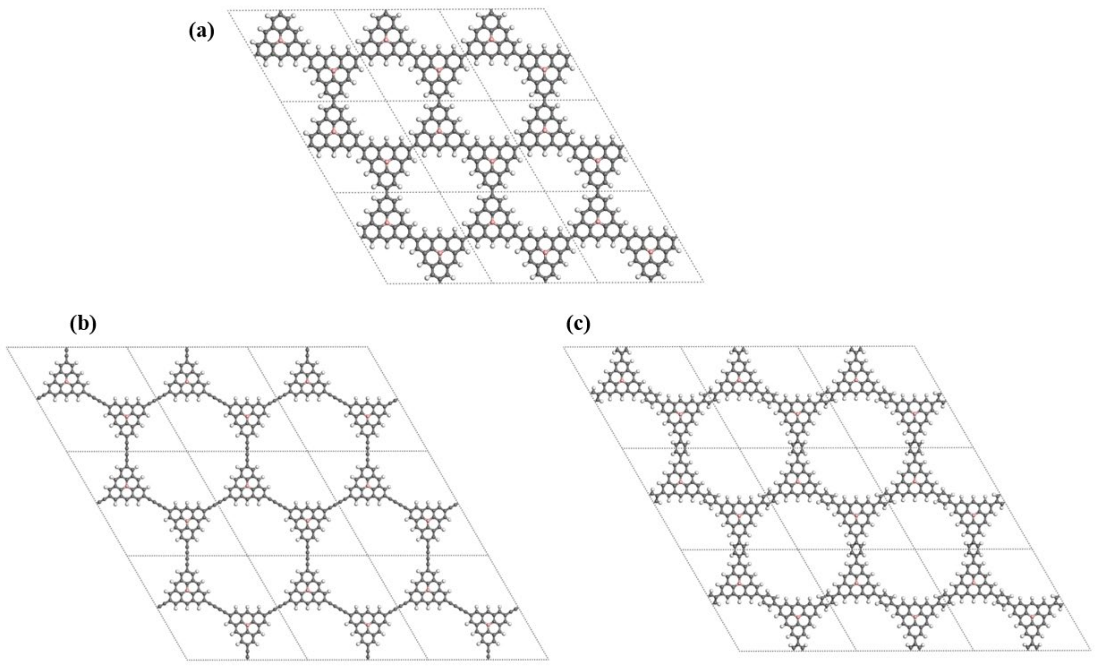

Fig. S3. Chemical structures of [TRI(B)], [TRI(B)-CCCC] and [TRI(B)-Ph]. (A)-(C) correspond to [TRI(B)], [TRI(B)-CCCC] and [TRI(B)-Ph], respectively.

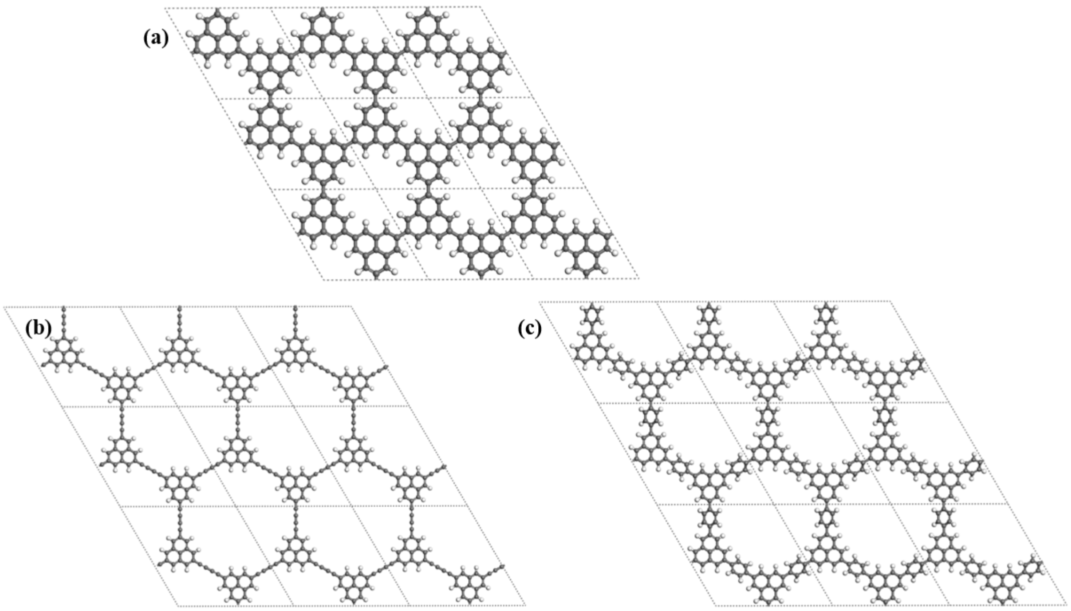

Fig. S4. Chemical structures of [PLY], [PLY-CCCC] and [PLY-Ph]. (A)-(C) correspond to [PLY], [PLY-CCCC] and [PLY-Ph], respectively.

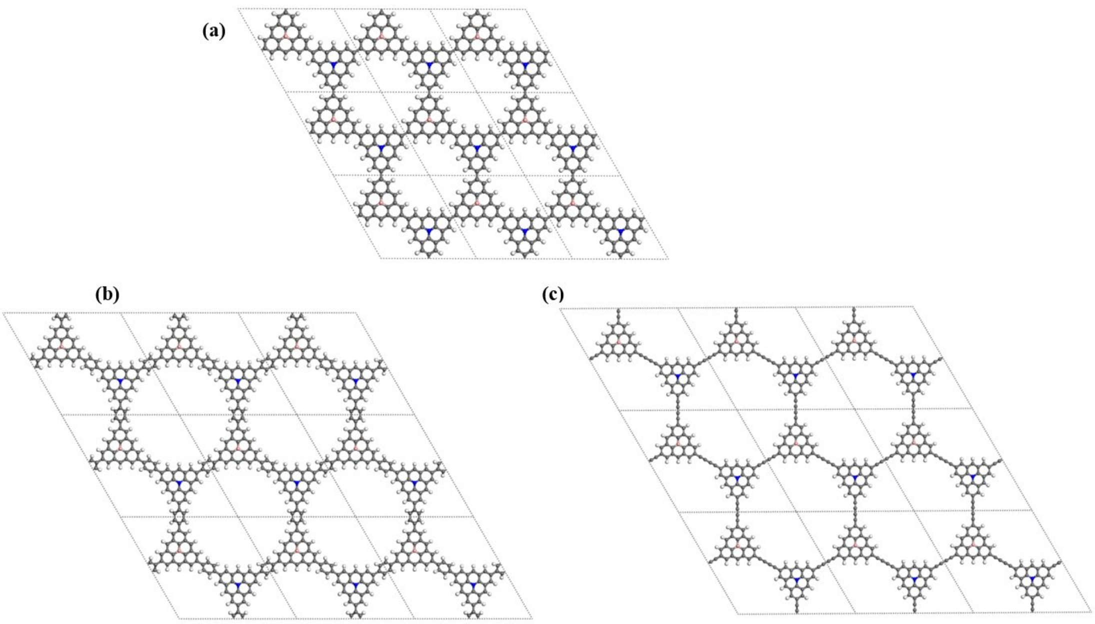

Fig. S5. Chemical structures of [TRI(B)-TRI(N)], [TRI(B)-CCCC-TRI(N)] and [TRI(B)-Ph-TRI(N)]. (A)-(C) correspond to [TRI(B)-TRI(N)], [TRI(B)-Ph-TRI(N)] and [TRI(B)-CCCC-TRI(N)], respectively.

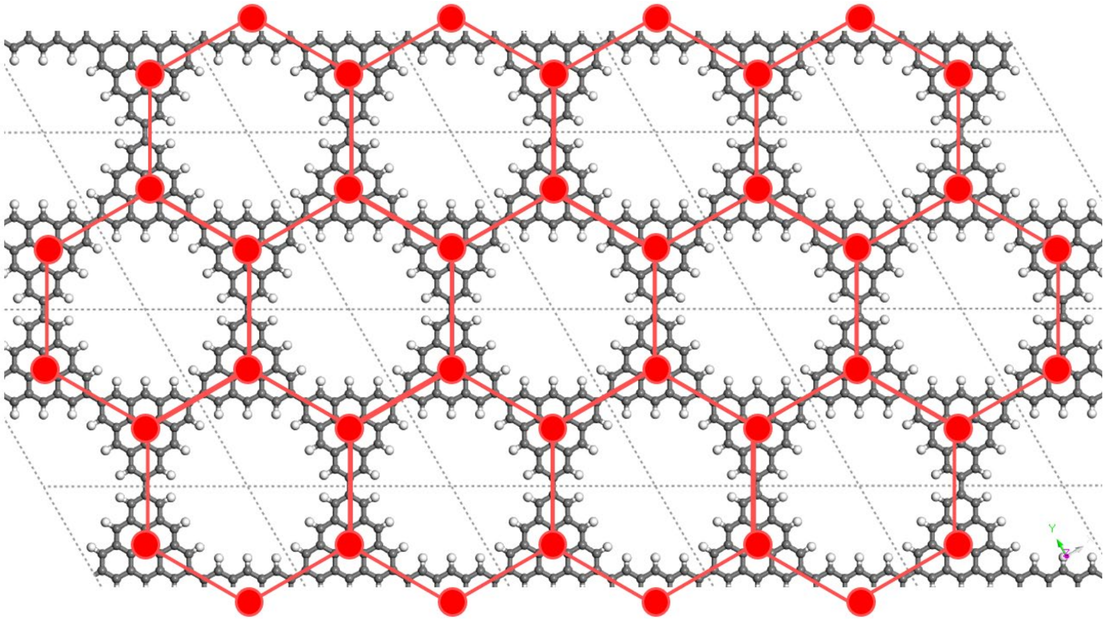

Fig. S6. Illustration of honeycomb lattice in triangulene 2D polymer.

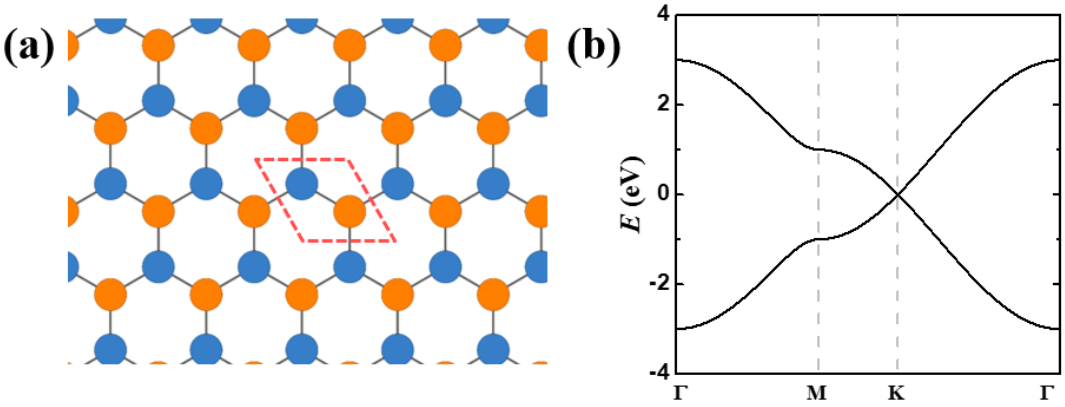

Fig. S7. The lattice structure and tight-binding band structure of honeycomb lattice with one orbital on each site. (A)-(B) correspond to the lattice structure and band structure, respectively.

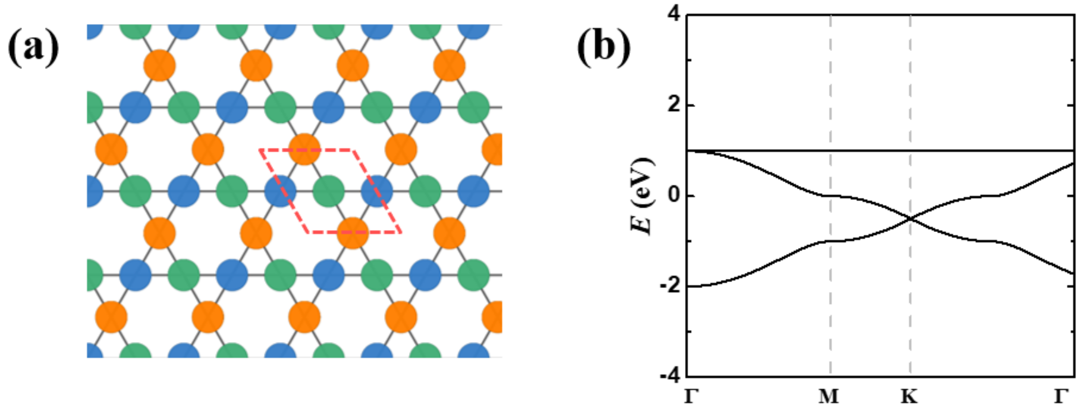

Fig. S8. The lattice structure and tight-binding band structure of kagome lattice. (A)-(B) correspond to the lattice structure and band structure, respectively.

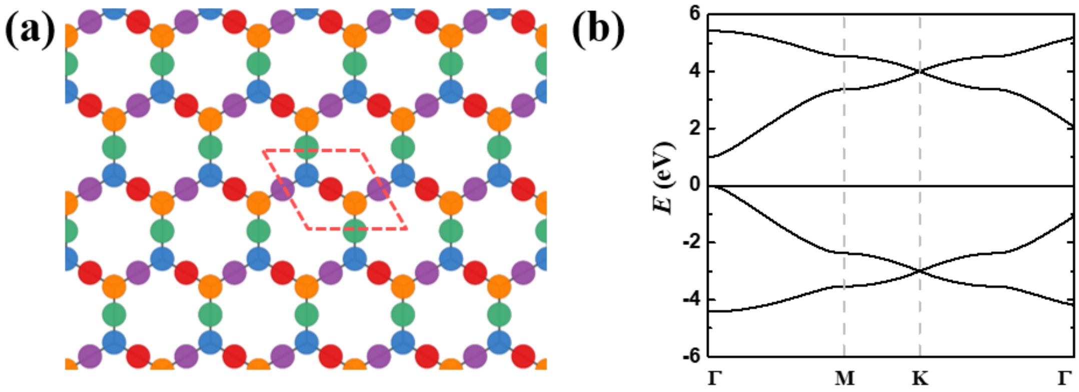

Fig. S9. The lattice structure and tight-binding band structure of honeycomb-kagome lattice. (A)-(B) correspond to structure and band structure, respectively.

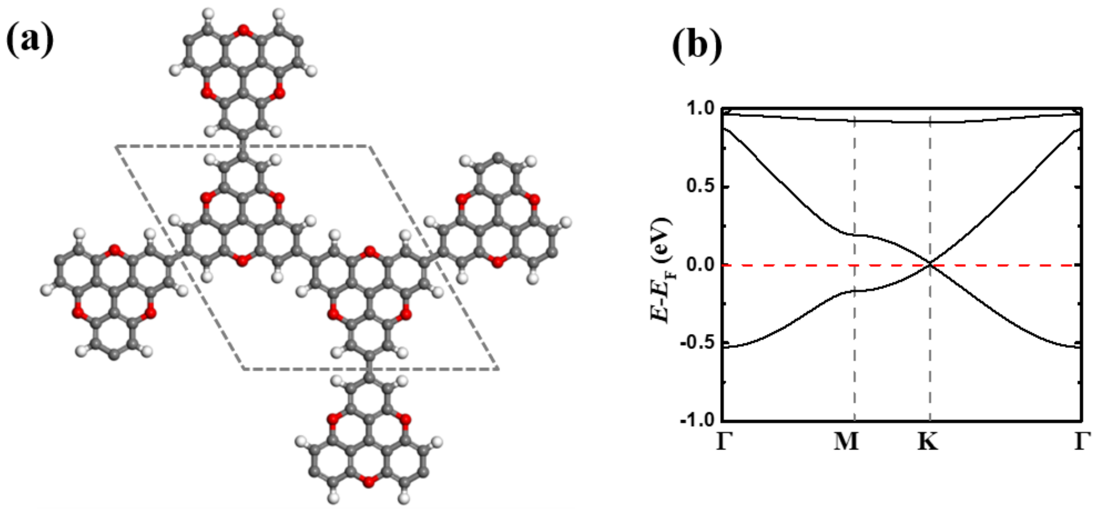

Fig. S10. Chemical structure and band structure of 2D polymer made of TAM for the diamagnetic state. (A)-(B) correspond to the chemical structure and band structure, respectively.

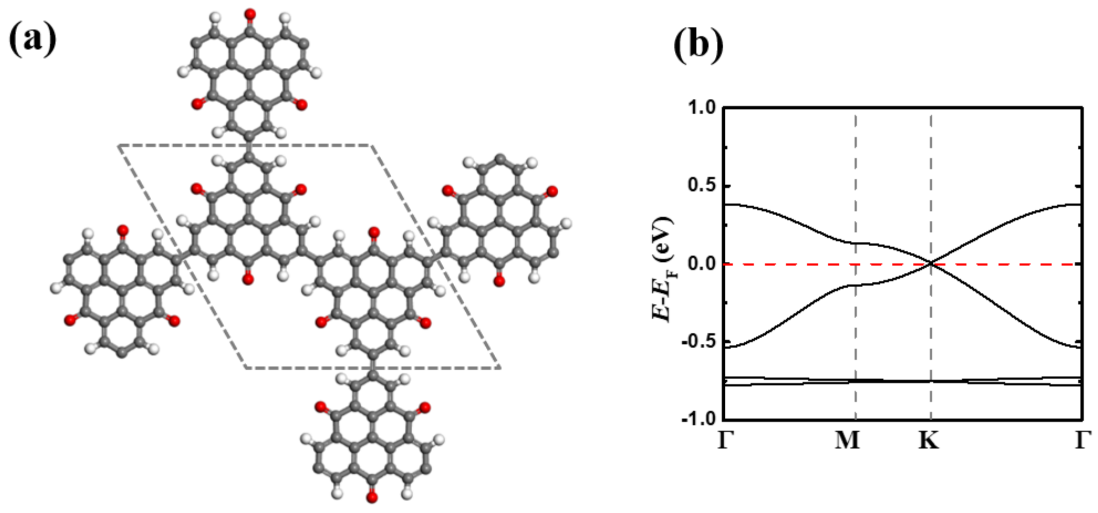

Fig. S11. Chemical structure and band structure of 2D polymer made of TOT for the diamagnetic state. (A)-(B) correspond to chemical structure and band structure, respectively.

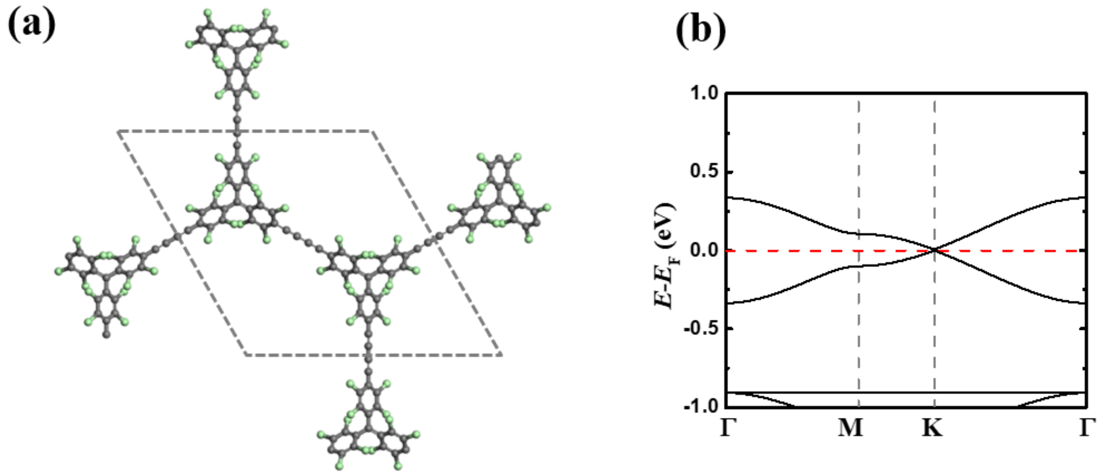

Fig. S12. Chemical structure and band structure of 2D polymer made of PTM for the diamagnetic state. (A) and (B) correspond to chemical structure and band structure, respectively.

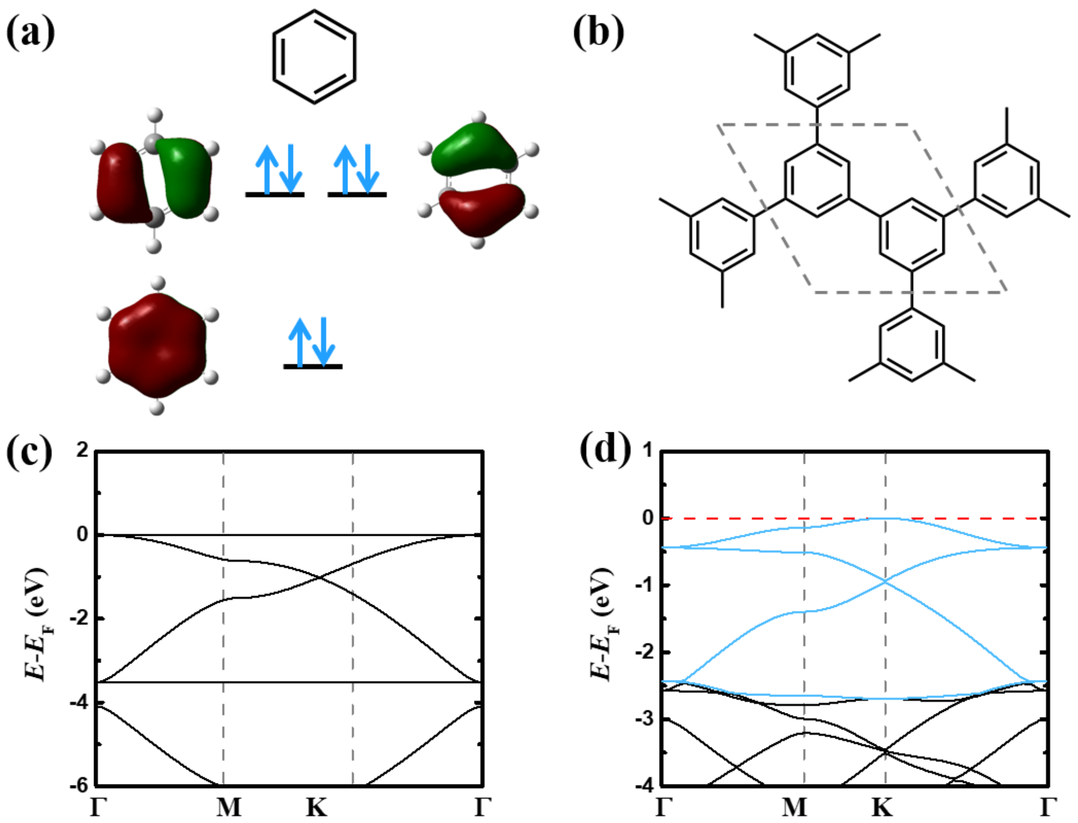

Fig. S13. Electronic structures of the benzene molecule and its 2D polymer. (A) Frontier p-orbitals of benzene molecule. (B) Structure illustration of 2D polymer made of benzene. Band structures of this 2D polymer calculated at tight-binding level (C) and PBE level (D).

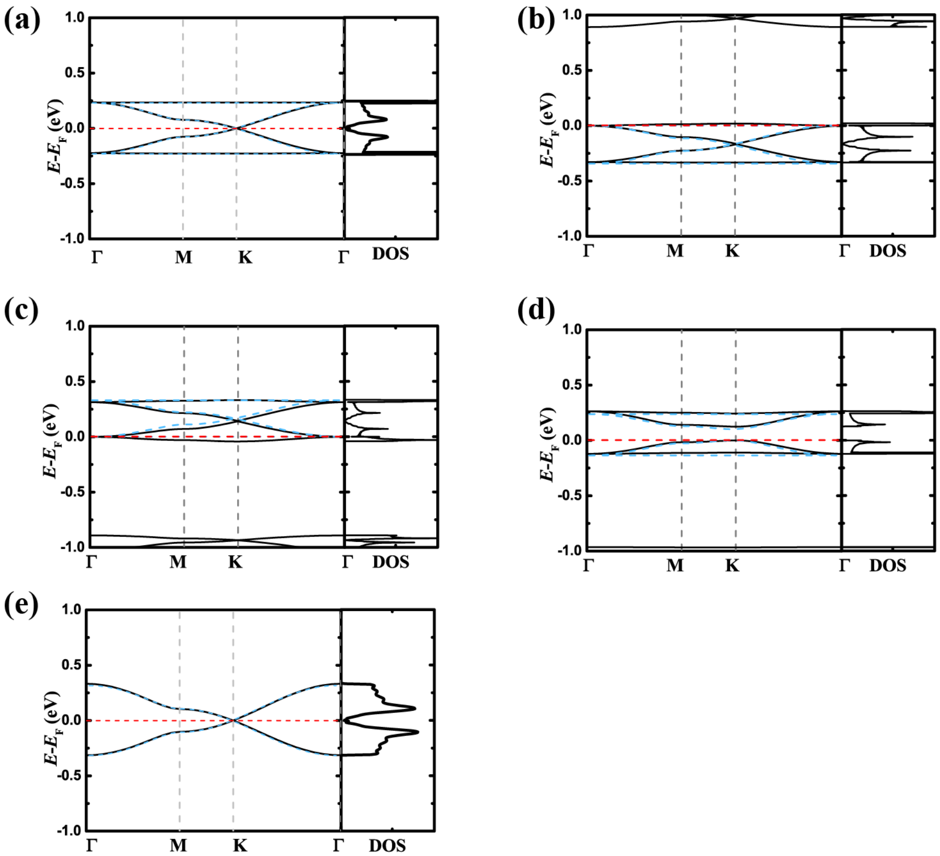

Fig. S14. Band structures and density of states of the diamagnetic state for triangulene-based 2D polymers at PBE0 level. (A)-(E) correspond to [TRI], [TRI(N)], [TRI(B)], [TRI(B)-TRI(N)], and [PLY], respectively. The blue dash lines are calculated by the tight-binding model. The red lines indicate the Fermi level.

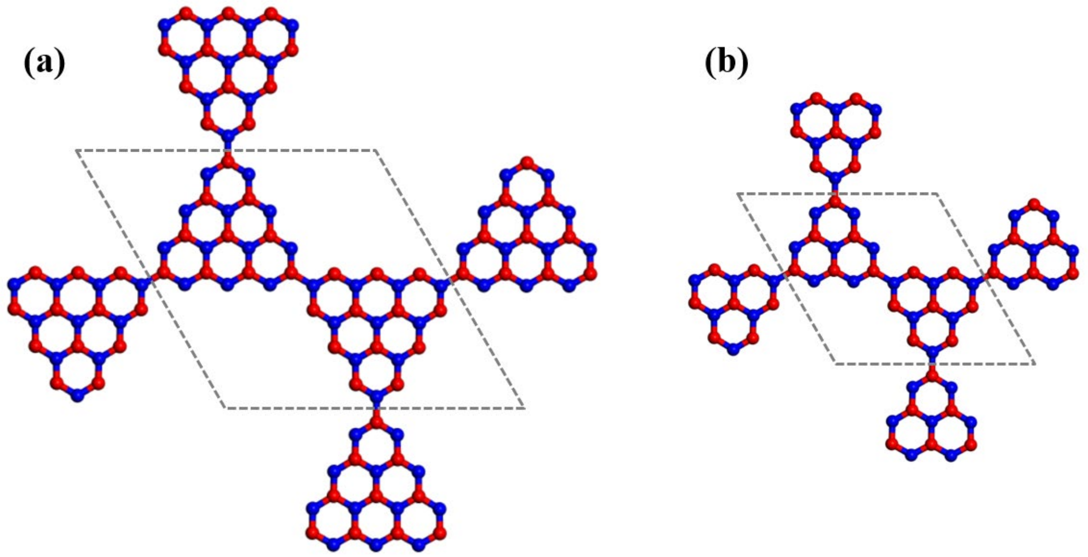

Fig. S15. Illustration of balanced sublattices in [TRI] and [PLY] according to the Ovchinnikov's rule. (A) and (B) correspond to [TRI] and [PLY], respectively. A and B sublattices are shown in blue and red, respectively.

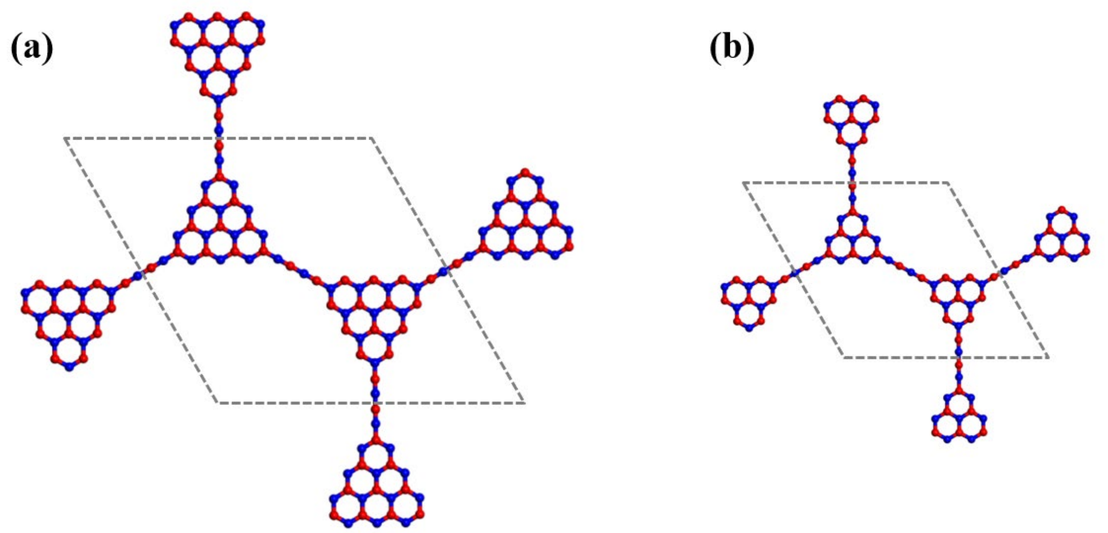

Fig. S16. Illustration of balanced sublattices in [TRI-CCCC] and [PLY- CCCC] according to the Ovchinnikov's rule. (A) and (B) correspond to [TRI-CCCC] and [PLY- CCCC], respectively. A and B sublattices are shown in blue and red, respectively.

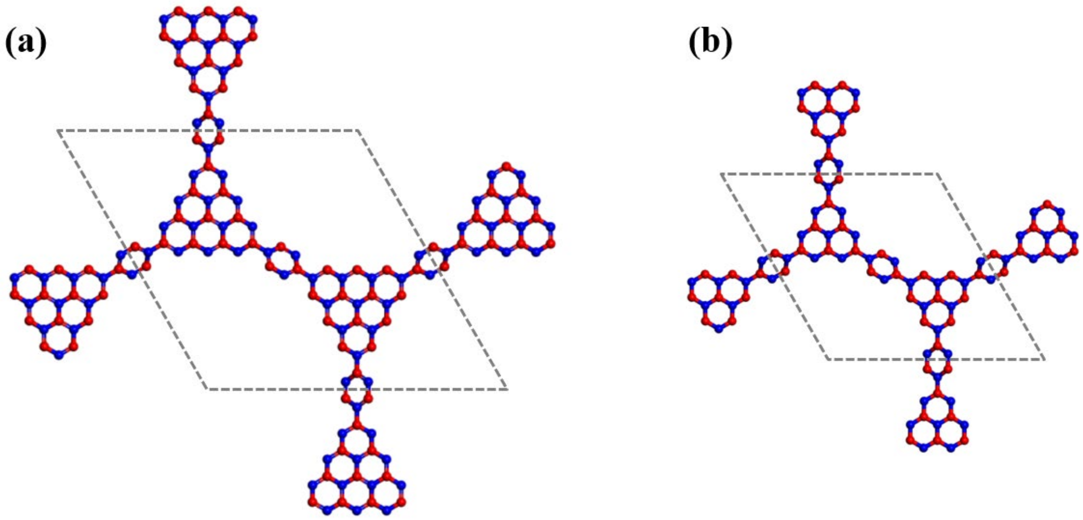

Fig. S17. Illustration of balanced sublattices in [TRI-Ph] and [PLY-Ph] according to the Ovchinnikov's rule. (A) and (B) correspond to [TRI-Ph] and [PLY-Ph], respectively. A and B sublattices are shown in blue and red, respectively.

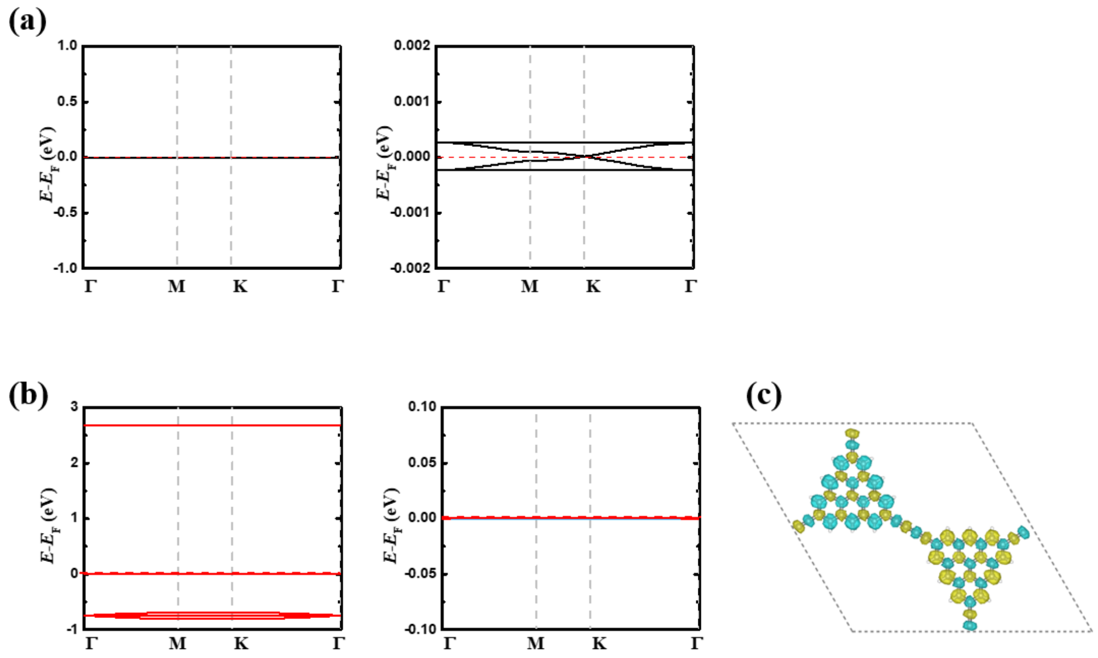

Fig. S18. Band structures of [TRI-CCCC] for the diamagnetic state and AFM state as well as the spin density distribution at the PBE0 level. (A) and (B) correspond to the band structures of the diamagnetic and AFM state. A different energy scale is used on the right to clearly depict the dispersion. (C) corresponds to the spin density distribution of [TRI-CCCC].

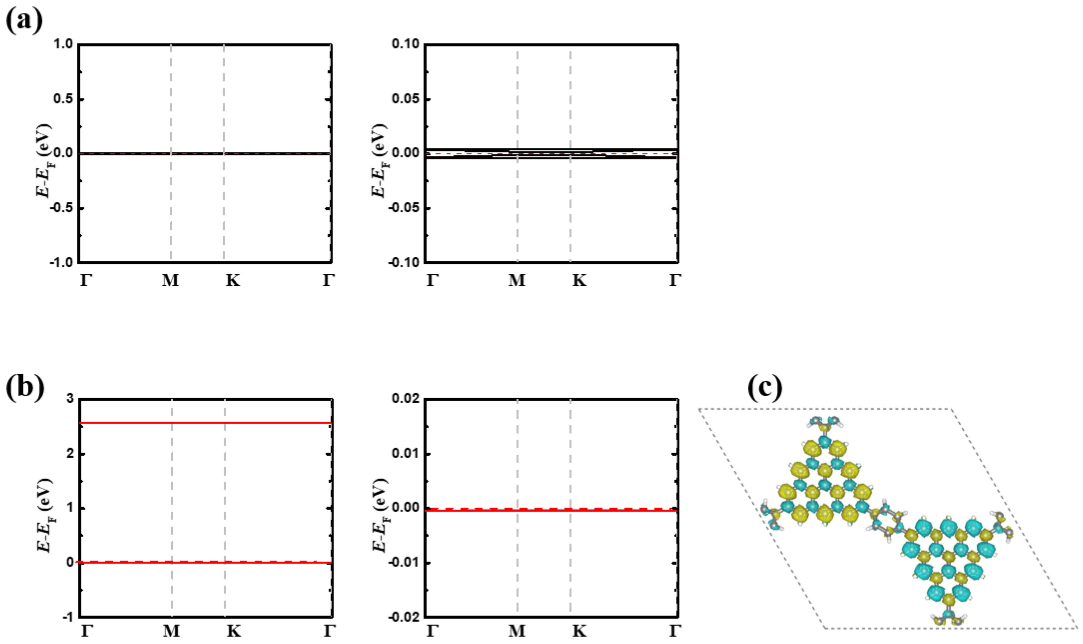

Fig. S19. Band structures of [TRI-Ph] for the diamagnetic state and AFM state as well as the spin density distribution at the PBE0 level. (A) and (B) correspond to the band structures of the diamagnetic and AFM state. A different energy scale is used on the right to clearly depict the dispersion. (C) corresponds to the spin density distribution of [TRI-Ph].

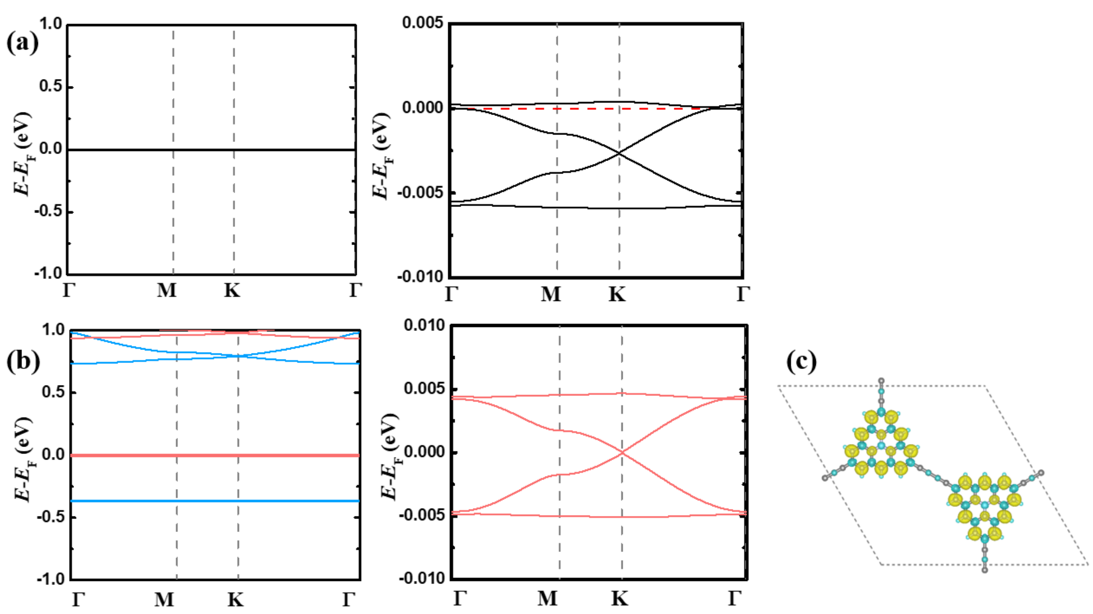

Fig. S20. Band structures of [TRI(N)-CCCC] for the diamagnetic state and FM state as well as the spin density distribution at the PBE level. (A) and (B) correspond to the band structures of the diamagnetic and FM state. A different energy scale is used on the right to clearly depict the dispersion. (C) corresponds to the spin density distribution of [TRI(N)-CCCC].

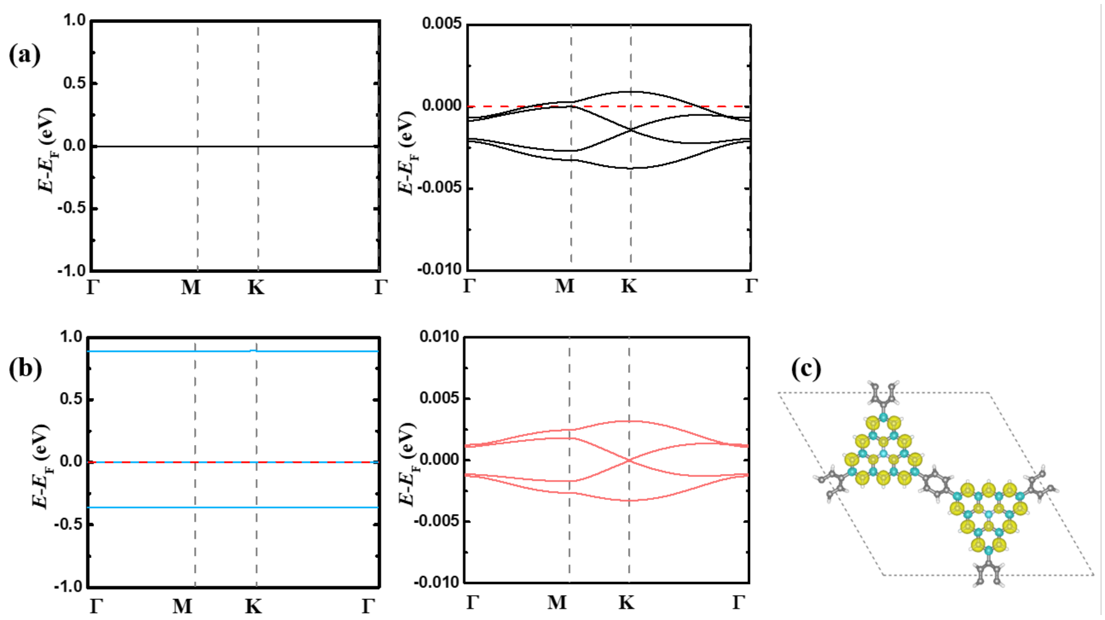

Fig. S21. Band structures of [TRI(N)-Ph] for the diamagnetic state and FM state as well as the spin density distribution at the PBE level. (A) and (B) correspond to the band structures of the diamagnetic and FM state. A different energy scale is used on the right to clearly depict the dispersion. (C) corresponds to the spin density distribution of [TRI(N)-Ph].

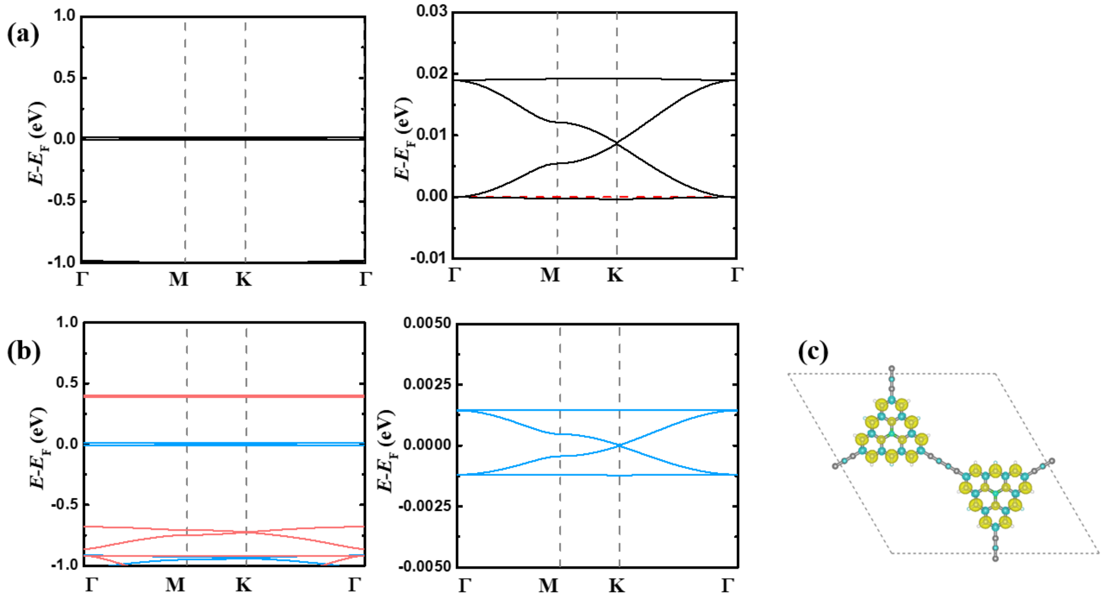

Fig. S22. Band structures of [TRI(B)-CCCC] for the diamagnetic state and FM state as well as the spin density distribution at the PBE level. (A) and (B) correspond to the band structures of the diamagnetic and FM state. A different energy scale is used on the right to clearly depict the dispersion. (C) corresponds to the spin density distribution of [TRI(B)-CCCC].

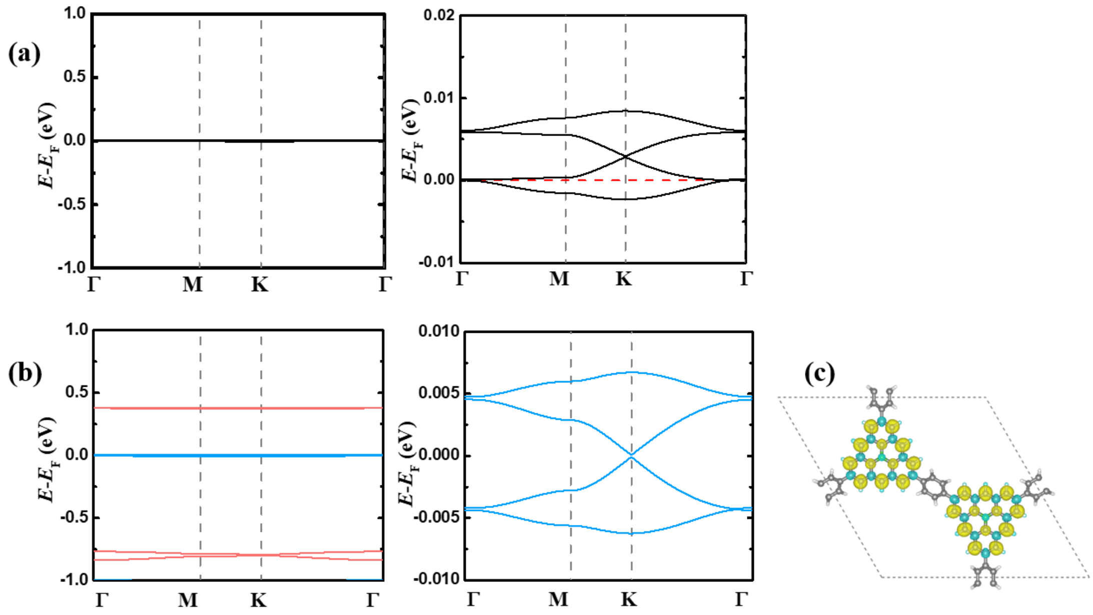

Fig. S23. Band structures of [TRI(B)-Ph] for the diamagnetic state and FM state as well as the spin density distribution at the PBE level. (A) and (B) correspond to the band structures of the diamagnetic and FM state. A different energy scale is used on the right to clearly depict the dispersion. (C) corresponds to the spin density distribution of [TRI(B)-Ph].

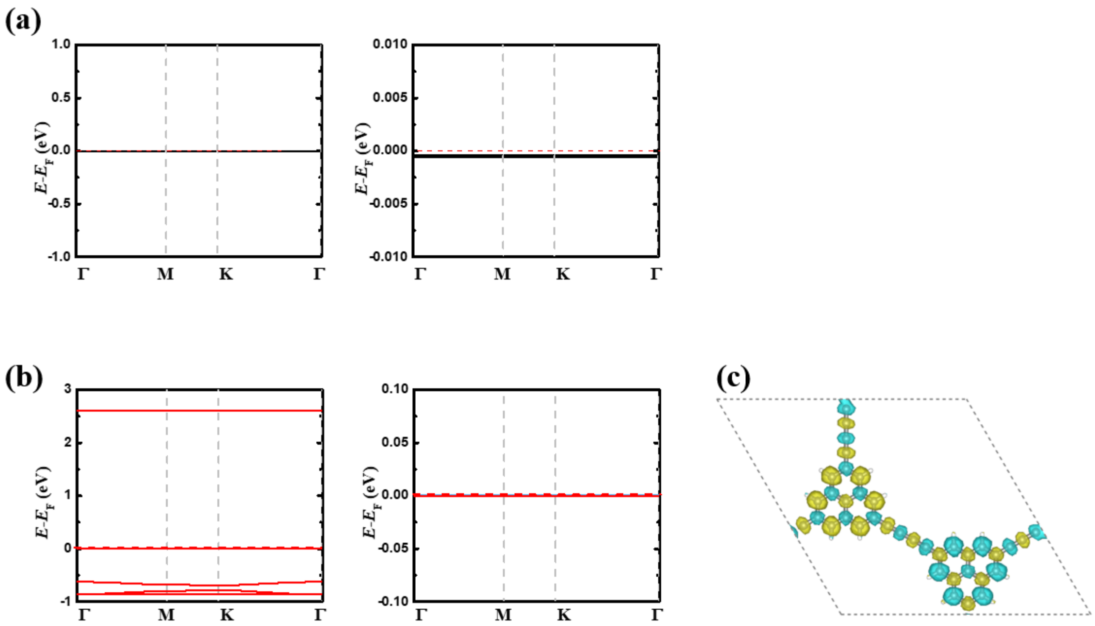

Fig. S24. Band structures of [PLY-CCCC] for the diamagnetic state and AFM state as well as the spin density distribution at the PBE0 level. (A) and (B) correspond to the band structures of the diamagnetic and AFM state. A different energy scale is used on the right to clearly depict the dispersion. (C) corresponds to the spin density distribution of [PLY-CCCC].

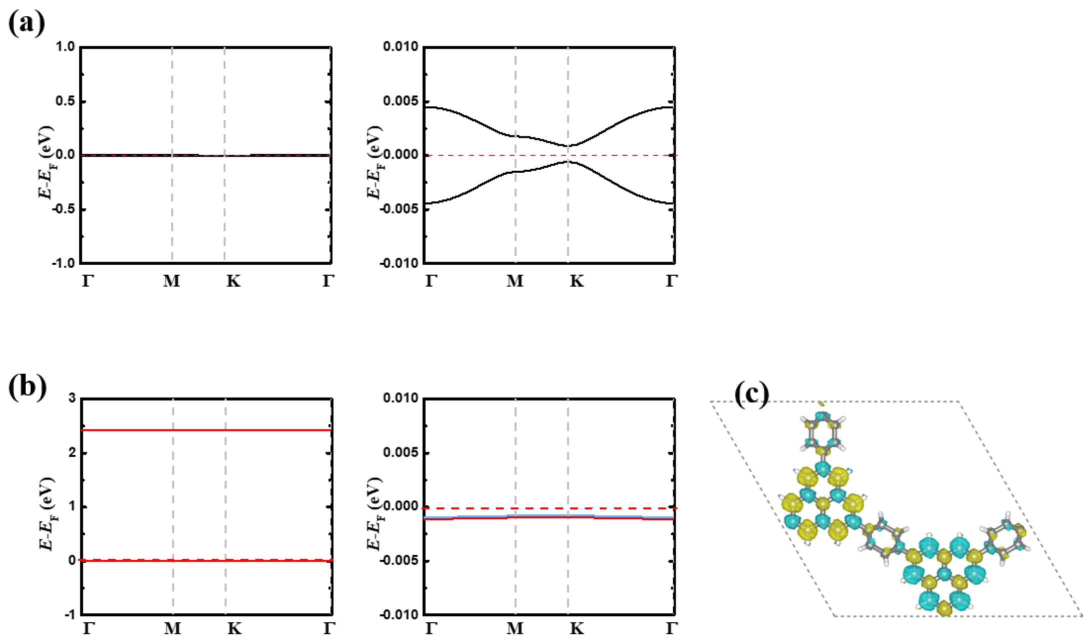

Fig. S25. Band structures of [PLY-Ph] for the diamagnetic state and AFM state as well as the spin density distribution at the PBE0 level. (A) and (B) correspond to the band structures of the diamagnetic and AFM state. A different energy scale is used on the right to clearly depict the dispersion. (C) corresponds to the spin density distribution of [PLY-Ph].

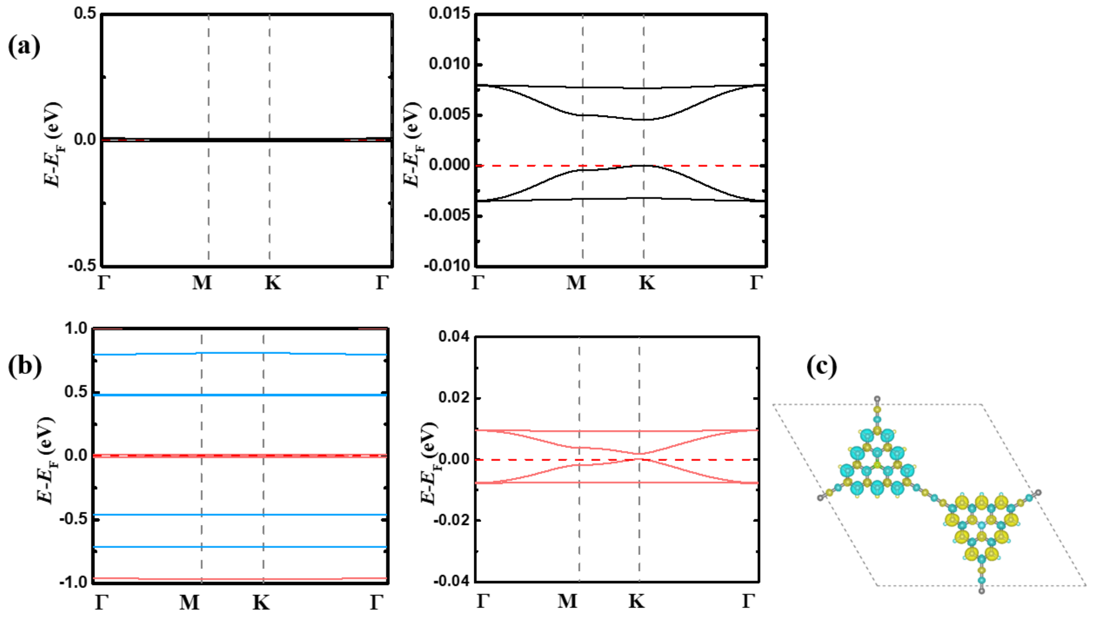

Fig. S26. Band structures of [TRI(B)-CCCC-TRI(N)] for the diamagnetic state and AFM state as well as the spin density distribution at the PBE level. (A) and (B) correspond to the band structures of the diamagnetic and AFM state. A different energy scale is used on the right to clearly depict the dispersion. (C) corresponds to the spin density distribution of [TRI(B)-CCCC-TRI(N)].

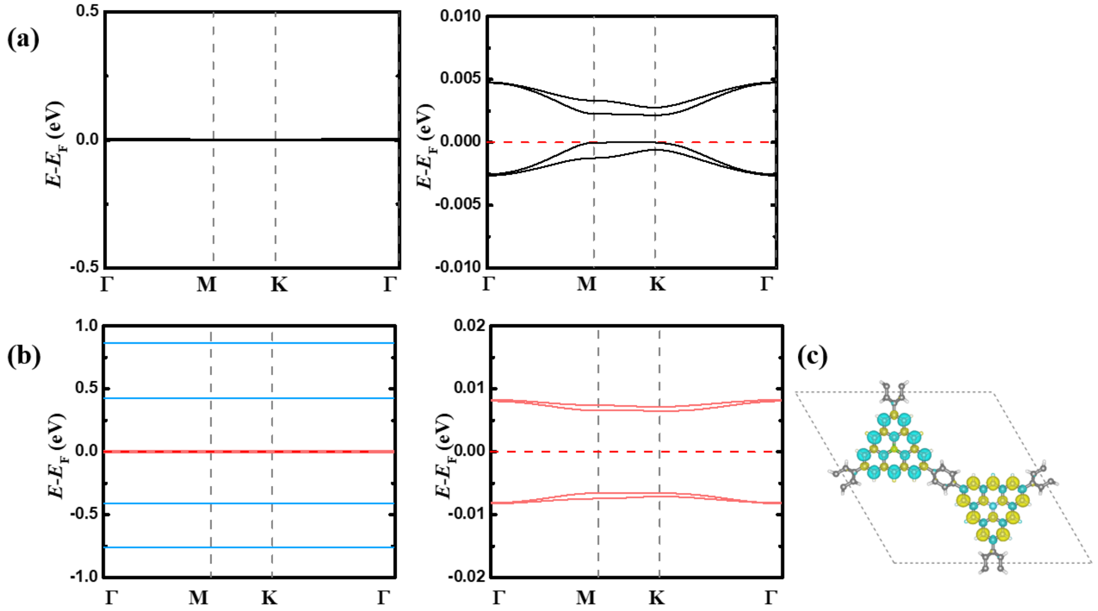

Fig. S27. Band structures of [TRI(B)-Ph-TRI(N)] for the diamagnetic state and AFM state as well as the spin density distribution at PBE level. (A) and (B) correspond to the band structures of the diamagnetic and AFM state. A different energy scale is used on the right to clearly depict the dispersion. (C) corresponds to the spin density distribution of [TRI(B)-Ph-TRI(N)].

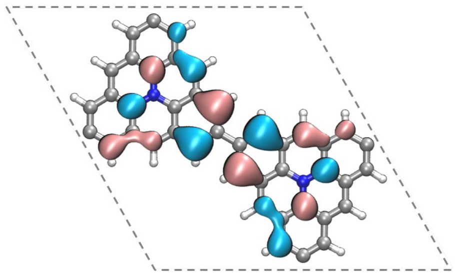

Fig. S28. Wavefunction for the down-spin of [TRI(N)] at the Dirac point.

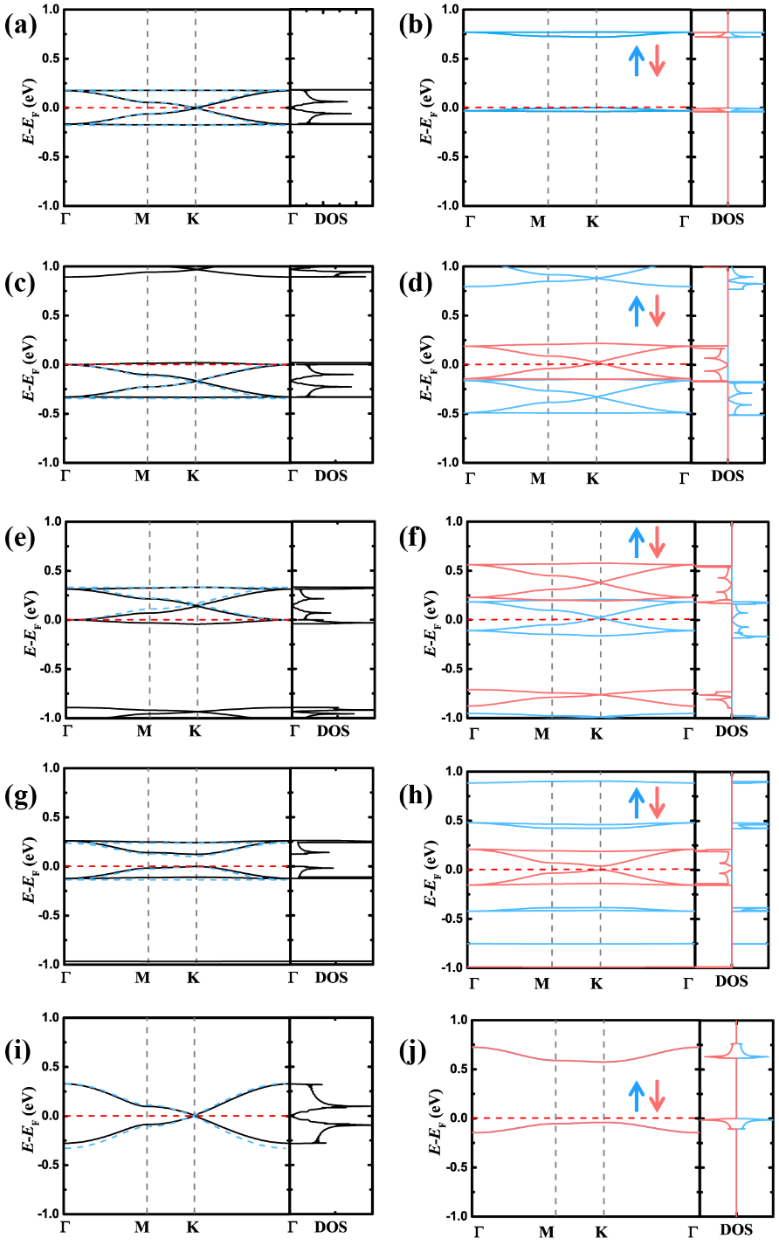

Fig. S29. Band structure and density of states for the diamagnetic and the spin-polarized ground state of triangulene-based 2D polymers calculated at the PBE level. (A), (C), (E), (G,) (I) correspond to the diamagnetic state. (B), (D), (F), (H), (J) correspond to the spin-polarized ground state. (A) and (B) correspond to [TRI]. (C) and (D) correspond to [TRI(N)]. (E) and (F) correspond to [TRI(B)]. (G) and (H) correspond to [TRI(B)-TRI(N)]. (I) and (J) correspond to [PLY]. For [TRI], [PLY], [TRI(B)-TRI(N)], the ground states are AFM states, while they are FM states for [TRI(N)] and [TRI(B)].

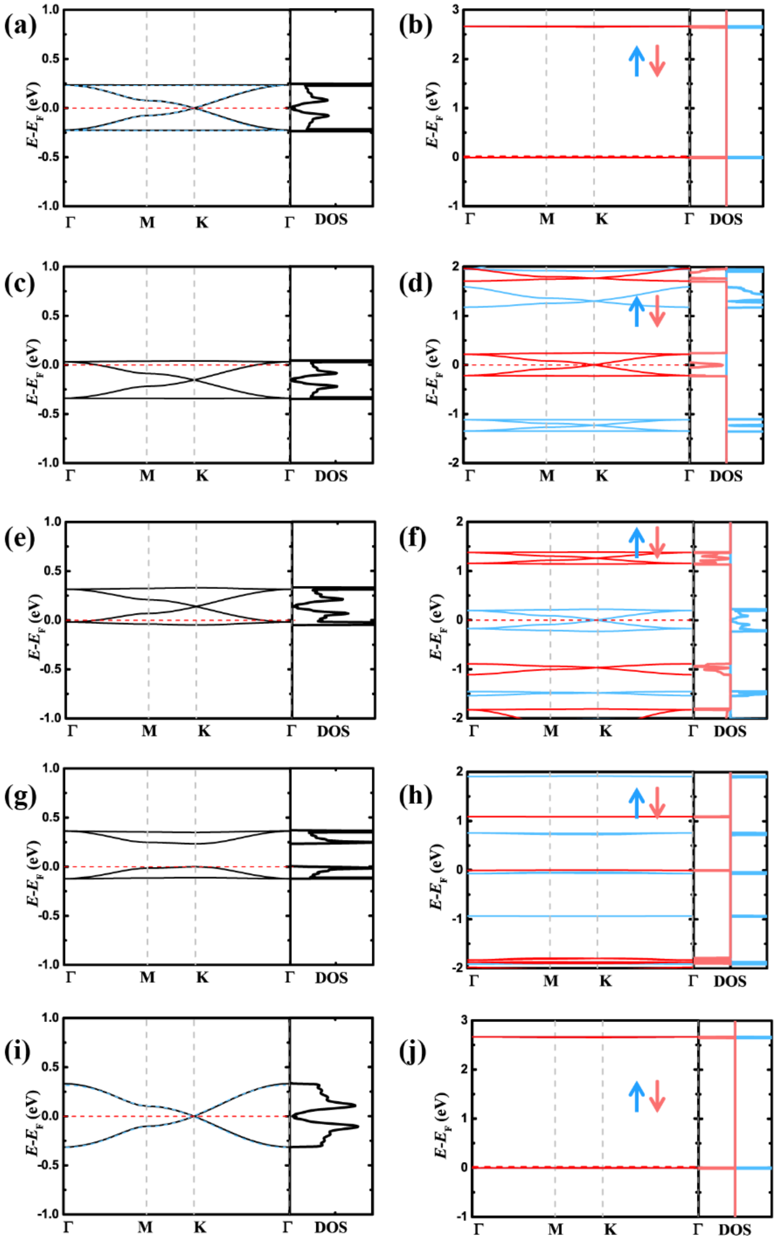

Fig. S30. Band structure and density of states for the diamagnetic and the spin-polarized ground state of triangulene-based 2D polymers calculated at the PBE0 level. (A), (C), (E), (G,) (I) correspond to the diamagnetic state. (B), (D), (F), (H), (J) correspond to the spin-polarized ground state. (A) and (B) correspond to [TRI]. (C) and (D) correspond to [TRI(N)]. (E) and (F) correspond to [TRI(B)]. (G) and (H) correspond to [TRI(B)-TRI(N)]. (I) and (J) correspond to [PLY]. For [TRI], [PLY], [TRI(B)-TRI(N)], the ground states are AFM states, while they are FM states for [TRI(N)] and [TRI(B)].

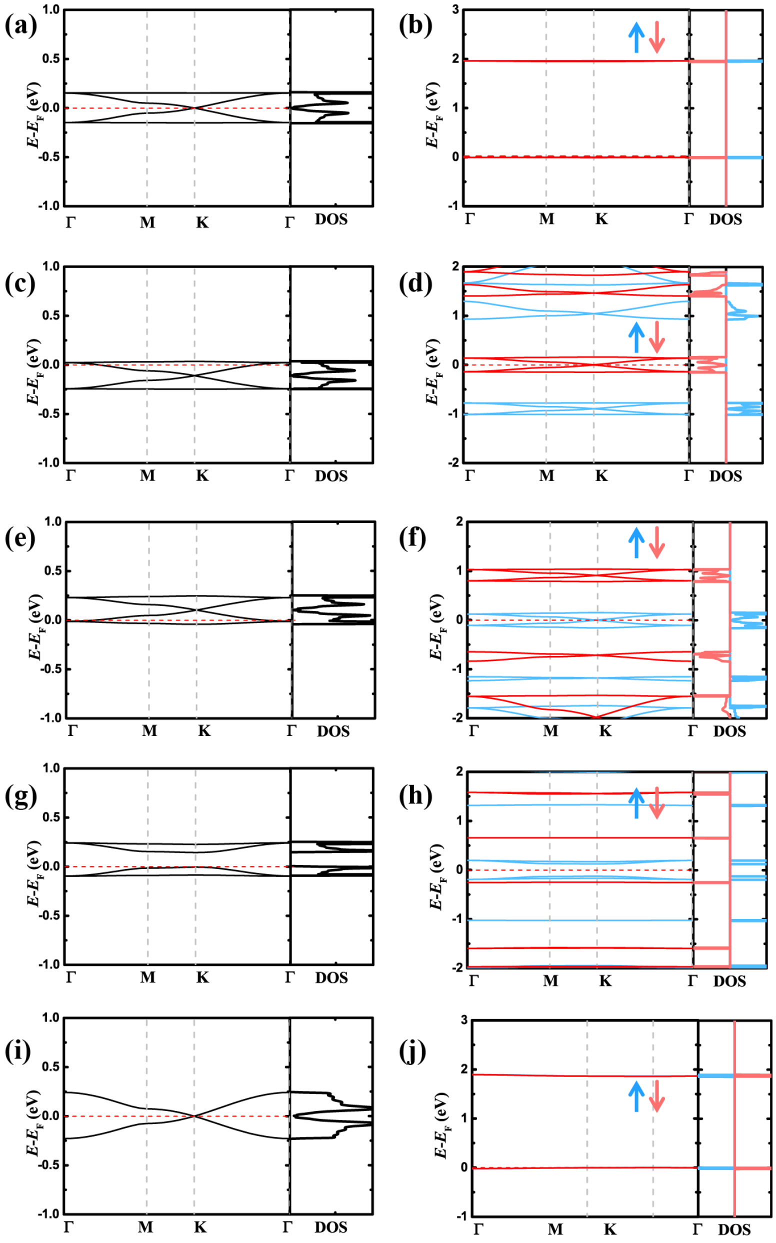

Fig. S31. Band structure and density of states of the diamagnetic and the spin-polarized ground state of triangulene-based 2D polymers calculated at the HSE06 level. (A), (C), (E), (G,) (I) correspond to the diamagnetic state. (B), (D), (F), (H), (J) correspond to the spin-polarized ground state. (A) and (B) correspond to [TRI]. (C) and (D) correspond to [TRI(N)]. (E) and (F) correspond to [TRI(B)]. (G) and (H) correspond to [TRI(B)-TRI(N)]. (I) and (J) correspond to [PLY]. For [TRI], [PLY], [TRI(B)-TRI(N)], the ground states are AFM states, while they are FM states for [TRI(N)] and [TRI(B)].

Table S1. Relative energies (*E* (meV)) of ferromagnetic (FM) ground state, antiferromagnetic state (AFM) and diamagnetic closed-shell state (CSS) for [TRI(N)] and [TRI(B)], calculated with PBE, PBE0 and HSE06 functionals.

		PBE	PBE0	HSE06
[TRI(N)]	FM	0	0	0
	AFM	89	163	102
	CSS	91	566	388
[TRI(B)]	FM	0	0	0
	AFM	84	141	88
	CSS	93	579	394

Table S2. Magnetic couplings *J* and spin-polarization energy ΔE_{spin} calculated at the PBE, PBE0 and HSE06 levels.

		PBE	PBE0	HSE06
[TRI]	J (meV)	-25	-32	-30
	ΔE_{spin} (eV)	-0.35	-2.17	-1.60
[TRI(N)]	J (meV)	59	109	68
	ΔE_{spin} (eV)	-0.09	-0.57	-0.39
[TRI(B)]	J (meV)	56	95	58
	ΔE_{spin} (eV)	-0.09	-0.58	-0.39
[TRI(B)-TRI(N)]	J (meV)	-125	-144	-186
	ΔE_{spin} (eV)	-0.07	-1.00	-0.65
[PLY]	J (meV)	-76	-98	-90
	ΔE_{spin} (eV)	-0.12	-1.04	-0.74

Table S3. Magnetic couplings (*J* (meV)) of [TRI(N)] and [TRI(B)] and relative energies (*E* (meV) per unit cell) calculated with CP2K package. It includes of the closed-shell singlet state (CSS) and the FM high-spin state (HS) and the AFM open-shell singlet state (OSS). The OSS states are calculated by restricted open-shell Kohn-Sham DFT (ROKS). The CSS and HS states are calculated by restricted DFT (RDFT) and unrestricted DFT (UDFT), respectively. PBE functional with DZVP-MOLOPT basis set and Goedecker-Teter-Hutter (GTH) pseudopotential are used.

E (meV)	[TRI(N)]	[TRI(B)]
J	66.3	69.1
CSS	0.0	0.0
OSS	-7.9	-19.2
HS	-107.4	-122.8
